# Pilot Assessment of Impacts of Ozone and Ozone/Hydrogen Peroxide Treatment on the Fate of Per- and Polyfluoroalkyl Substances and Precursors

**DOI:** 10.1021/acsestwater.4c00565

**Published:** 2024-09-25

**Authors:** Xiaoyue Xin, Juhee Kim, ShihChi Weng, Ching-Hua Huang

**Affiliations:** † School of Civil and Environmental Engineering, Georgia Institute of Technology, Atlanta, Georgia 30332, United States; ‡ Department of Civil, Environmental and Construction Engineering, University of Hawaìi at Ma̅noa, Honolulu, Hawaii 96822, United States; § Department of Water Resources, Gwinnet County Government, Lawrenceville, Georgia 30045, United States

**Keywords:** short-chain PFAS, perfluorinated alkyl acids (PFAA) precursors, ozone, ozone/H_2_O_2_ advanced oxidation process, wastewater reuse, drinking water treatment

## Abstract

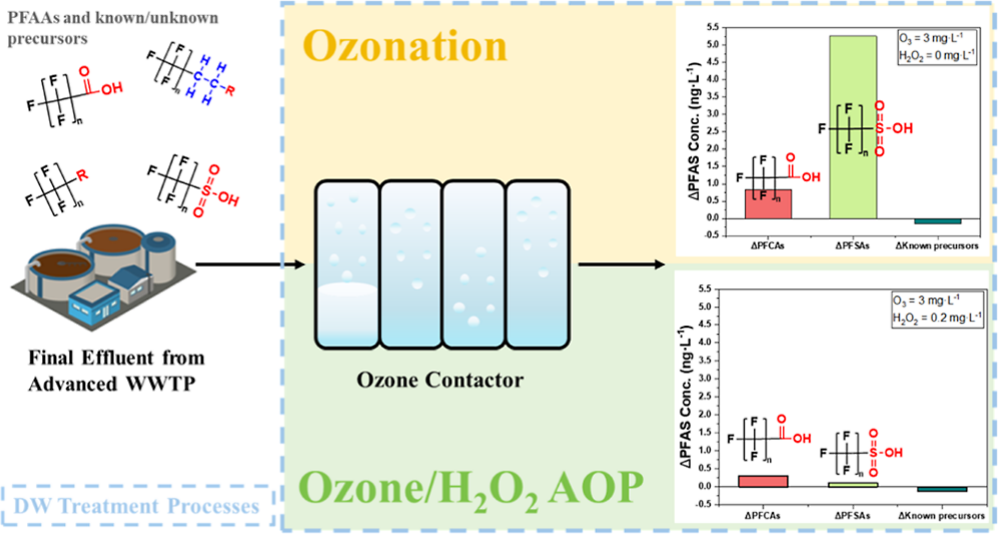

Per- and polyfluoroalkyl substances (PFAS) make up a large class of anthropogenic micropollutants prevalent in wastewater. Oxidative processes commonly used in wastewater potable reuse treatment may affect transformation of PFAS precursors, leading to elevated concentrations of perfluorinated alkyl acids (PFAAs) that are significant health concerns. This work conducted a pilot-scale investigation to assess the influence of ozonation (O_3_) and ozone/hydrogen peroxide (O_3_/H_2_O_2_) advanced oxidation process (AOP), respectively, on the fate of PFAS in a wastewater effluent subjected to reuse. The study evaluated 40 target PFAS and associated precursors [based on the total oxidizable precursor (TOP) assay] under various treatment conditions, including different ozone doses (1.0–4.0 mg·L^–1^), H_2_O_2_ doses (0–0.20 mg·L^–1^), and contact time (0–20 min). Results indicated that short-chain (C3–C7) PFAAs dominated in concentrations, while overall PFAA concentrations were elevated by both oxidative treatment processes, particularly after high-dose ozonation treatment. TOP assays revealed that there were considerable amounts of PFAA precursors in the reuse wastewater, and their concentrations were decreased after the oxidative treatment with an increase of some of the PFAAs. This pilot study demonstrated that ozone and ozone-based AOP treatments can have a moderate influence on the transformation of PFAS and increase in PFAA levels under practical conditions.

## Abstract 2

Advanced treatment of wastewater effluent by ozonation and ozone/H_2_O_2_ for potable reuse was investigated at a pilot scale and indicated the potential to increase transformation of PFAS/precursors and concentrations of perfluorinated alkyl acids.

## 1 Introduction

Per- and polyfluoroalkyl substances (PFASs) are a class of anthropogenic organofluorine compounds which pose significant health and ecological concerns because of their environmental persistence and toxicity.^1−3^ PFASs have been used in a large variety of industrial and consumer products such as firefighting foams, water-repellent fabrics, carpets, food packaging, and nonstick cookware.^4,5^ The disposal of PFAS-containing products and discharge of contaminated wastewaters result in the omnipresence of these compounds in the environment,^3,6^ tissues of wildlife,^7,8^ and even human blood and breast milks.^3,9−11^ Recognizing the risks associated with PFAS, the U.S. Environmental Protection Agency (EPA) announced the final National Primary Drinking Water Regulation for six PFASs on April 10, 2024, including perfluorooctanoic acid (PFOA), perfluorooctanesulfonic acid (PFOS), perfluorobutanesulfonic acid (PFBS), perfluorohexanesulfonic acid (PFHxS), perfluorononanoic acid (PFNA), and hexafluoropropylene oxide dimer acid (HFPO–DA or so-called GenX). EPA established the enforceable levels [or maximum contaminant levels (MCLs)] in drinking water for five individual PFAS: PFOA and PFOS at 4 ng·L^–1^, respectively; PFHxS, PFNA, and HFPO–DA at 10 ng·L^–1^, respectively; and for PFAS mixtures containing at least two or more of PFHxS, PFNA, HFPO–DA, and PFBS, a calculated hazard index (HI) not exceeding 1.0 according to eq 1.^12^
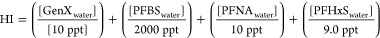
1

Most PFASs are highly recalcitrant to conventional wastewater treatment processes, and thus wastewater treatment plants (WWTPs) have become significant point sources of PFAS to the aquatic environment.^13−16^ Hence, the risk of PFAS entering drinking water from the potable reuse of wastewater is also of particular concern. Currently, there are various advanced treatment processes used for potable reuse systems after typical wastewater treatment including membrane filtration, adsorption, ion exchange, and foam fractionation. One of the most common treatment technologies is to apply reverse osmosis (RO), which is a well-established membrane technology and has been proven to be highly effective at reducing the concentrations of a wide variety of dissolved contaminants.^17−19^ However, RO-based treatment trains are capital and operationally cost-intensive, particularly for inland facilities where the brine concentrates cannot be disposed of through marine discharge.^19,20^ These limitations have driven research toward the investigation of alternative technologies for avoiding generation of waste concentrates that are challenging for inland disposal and for reducing treatment costs. In such a group, advanced oxidation technologies (AOTs) such as ozonation (O_3_),^21−23^ peroxonation (O_3_/H_2_O_2_),^24,25^ and UV-driven advanced oxidation processes (AOPs) (H_2_O_2_, Cl_2_)^23,25−29^ are being studied and implemented in full scale for advanced wastewater and drinking water treatment. The AOTs can be engineered alone, or in combination with biological and physical steps such as pre- and post-treatment with biological activated carbon (BAC).^30−34^ For example, ozonation combined with BAC has been used as part of an advanced wastewater treatment train to produce a high-quality drinking water source.^35^ However, some of these processes, like chlorination, ozonation, ozonation/H_2_O_2_, and UV-AOPs, may potentially transform PFAS precursors into terminal perfluorinated alkyl acids (PFAAs).^36−39^ Such a transformation could lead to an increase in the concentration of PFAAs in treated water, posing a challenge for meeting the stringent PFAS regulatory limits set by the U.S. EPA. Hence, more research is needed to better understand the impacts of AOTs on the fate of PFAS and precursors in advanced water treatment.

The overall objective of this study was to assess the influence of ozonation (O_3_) and ozone/hydrogen peroxide (O_3_/H_2_O_2_) AOP on the fate of PFAS in a wastewater effluent subjected to potable reuse. A wide range of known PFAS compounds (40 total), including short- and long-chain PFAAs and their precursors, were selected for monitoring. Additionally, unknown PFAS precursors were evaluated using the total oxidizable precursor (TOP) assay.^40−43^ This study was conducted at a pilot-scale drinking water treatment plant (DWTP), equipped with wastewater feed from an advanced WWTP and with ozonation, O_3_/H_2_O_2_, and other drinking water treatment processes, to evaluate the impacts of AOTs and PFAS transformation under real-time water treatment conditions. Water samples were collected from the pilot treatment trains at various oxidant dosages and contact times to investigate the trends in PFAS fate. The results were characterized and used to derive insight. Thus, far, pilot- or full-scale investigations of impacts of AOTs on the fate of PFAS are still limited. This study included a comprehensive evaluation of the fate of 40 known PFAS compounds, as well as unknown precursors. This pilot study adds to the data of a more realistic assessment of PFAS fate impacted by ozonation and O_3_/H_2_O_2_ and provides insights for current and future operations of AOTs regarding wastewater reuse and PFAS challenge.

## 2 Materials and Methods

### 2.1 Chemicals

40 PFASs (Table 1) were selected for monitoring: 11 PFCAs (PFBA, PFPeA, PFHxA, PFHpA, PFOA, PFNA, PFDA, PFUdA, PFDoA, PFTrDA, and PFTeDA); eight PFSAs (PFPrS, PFBS, PFPeS, PFHxS, PFHpS, PFOS, PFNS, and PFDS); and 21 “known precursors”: three fluorotelomer sulfonic acids (FTSs) (4:2 FTS, 6:2 FTS, and 8:2 FTS), three fluorotelomer (unsaturated) carboxylic acids [FT(U)Cas] (6:2 FTCA, 5:3 FTCA, and 6:2 FTUCA), three perfluorosulfonamides (FBSA, FHxSA, and PFOSA), two perfluorosulfonamidoacetic acids (N-MeFOSAA and N-EtFOSAA), eight per- and polyfluoroethers (HFPO–DA, ADONA, 9Cl-PF3ONS, 11Cl-PF3OUdS, PFEESA, PF4OPeA, PF5OHxA, and 3,6-OPFHpA), and two fluorotelomer phosphate diesters (6:2 diPAP and 6:2/8:2 diPAP). Sources of analytical standards and isotope-labeled surrogates used in this study are provided in Supporting Information Text S1. High purity deionized water (DI water, >18 mΩ cm) used in this study was generated from a Barnstead water purification system (Thermo Fisher Scientific).

**Table 1:** 40 PFAS Analyzed in This Study

acronym	chemical name	chemical formula
Perfluorocarboxylic Acids		
PFBA	perfluorobutanoic acid	C_3_F_7_CO_2_H
PFPeA	perfluoropentanoic acid	C_4_F_9_CO_2_H
PFHxA	perfluorohexanoic acid	C_5_F_11_CO_2_H
PFHpA	perfluoroheptanoic acid	C_6_F_13_CO_2_H
PFOA	perfluorooctanoic acid	C_7_F_15_CO_2_H
PFNA	PFNA	C_8_F_17_CO_2_H
PFDA	perfluorodecanoic acid	C_9_F_19_CO_2_H
PFUnA	perfluoroundecanoic acid	C_10_F_21_CO_2_H
PFDoA	perfluorododecanoic acid	C_11_F_23_CO_2_H
PFTrDA	perfluorortridecanoic acid	C_12_F_25_CO_2_H
PFTeDA	perfluorotetradecanoic acid	C_13_F_27_CO_2_H
Perfluorosulfonic Acids		
PFPrS	perfluoropropane sulfonic acid	C_3_F_7_SO_3_H
PFBS	perfluorobutane sulfonic acid	C_4_F_9_SO_3_H
PFPeS	perfluoropentane sulfonic acid	C_5_F_11_SO_3_H
PFHxS	perfluorohexane sulfonic acid	C_6_F_13_SO_3_H
PFHpS	perfluoroheptane sulfonic acid	C_7_F_15_SO_3_H
PFOS	perfluorooctane sulfonic acid	C_8_F_17_SO_3_H
PFNS	perfluorononane sulfonic acid	C_9_F_19_SO_3_H
PFDS	perfluorodecane sulfonic acid	C_10_F_21_SO_3_H
Fluorotelomer Sulfonic Acids		
4:2 FTS	4:2 FTS	C_6_H_4_F_9_SO_3_H
6:2 FTS	6:2 FTS	C_8_H_4_F_13_SO_3_H
8:2 FTS	8:2 FTS	C_10_H_4_F_17_SO_3_H
Fluorotelomer (Unsaturated) Carboxylic Acids		
6:2 FTCA	6:2 fluorotelomer carboxylic acid	C_6_F_13_CH_2_CO_2_H
5:3 FTCA	5:3 fluorotelomer carboxylic acid	C_5_CH_2_CH_2_F_11_CO_2_H
6:2 FTUCA	6:2 fluorotelomer unsaturated carboxylic acid	C_6_F_12_CHCO_2_H
Perfluorosulfonamides		
FBSA	perfluorobutane sulfonamide	C_4_F_9_SO_2_NH_2_
FHxSA	perfluorohexane sulfonamide	C_6_F_13_SO_2_NH_2_
PFOSA	perfluorooctane sulfonamide	C_8_F_17_SO_2_NH_2_
Perfluorosulfonamidoacetic Acids		
N-MeFOSAA	*N*-methyl-perfluoro-1-octanesulfonamidoacetic acid	C_8_F_17_SO_2_NHC_3_O_2_H_5_
N-EtFOSAA	*N*-ethyl-perfluoro-1-octanesulfonamidoacetic acid	C_8_F_17_SO_2_NHC_4_O_2_H_7_
Per- and Polyfluoroethers		
HFPO–DA	hexafluoropropylene oxide-dimer acid	C_3_F_7_OC_2_F_4_CO_2_H
ADONA	4,8-dioxa-3H-PFNA	CF_3_OC_3_F_6_OC_2_F_3_HCO_2_H
9Cl-PF3ONS	9-chlorohexadecafluoro-3-oxanone-1-sulfonic acid	ClC_6_F_12_OC_2_F_4_SO_3_H
11Cl-PF3OUdS	11-chloroeicosafluoro-3-oxaundecane-1-sulfonic acid	ClC_8_F_16_OC_2_F_4_SO_3_H
PFEESA	perfluoro(2-ethoxyethane)sulfonic acid	C_2_F_5_OC_2_F_4_SO_3_H
PF4OPeA	perfluoro-3-methoxypropanoic acid	CF_3_OC_2_F_4_CO_2_H
PF5OHxA	perfluoro-4-methoxybutanoic acid	CF_3_OC_3_F_6_CO_2_H
3,6-OPFHpA	nonafluoro-3,6-dioxaheptanoic acid	CF_3_OC_2_F_4_OCF_2_CO_2_H
Fluorotelomer Phosphate Diesters		
6:2 diPAP	6:2 fluorotelomer phosphate diester	C_6_F_13_C_2_H_4_PO_4_HC_2_H_4_C_6_F_13_
6:2/8:2 diPAP	6:2/8:2 fluorotelomer phosphate diester	C_6_F_13_C_2_H_4_PO_4_HC_2_H_4_C_8_F_17_

### 2.2 Pilot Plant Design and Sample Collection

The pilot facility (Figure S1a) is housed within a DWTP in Georgia. The pilot plant, at six gallons per minute (gpm) capacity, has a full treatment train that mimics the full-scale DWTP, including ozonation, coagulation/flocculation, direct filtration with anthracite/sand biofilters, and chlorine disinfection. In this study, only the ozonation process was utilized for ozone and ozone/H_2_O_2_ trials, as shown in Figure S1b. Instead of using the typical lake water source, the final effluent from an advanced WWTP [40 million gallons per day (mgd) running capacity, 60 MGD maximum capacity] was routed to the pilot plant and used as the sole source water, upstream of the inlet channel to the first treatment process (ozonation). Before entering the pilot plant, the wastewater was treated at the WWTP by primary treatment, activated sludge biological treatment, ultrafiltration and granular media filtration, preozonation, BAC filtration, and postozonation. The wastewater effluent did not contain residual ozone and had water quality characteristics as follows: pH = 6.0, turbidity = 0.1 NTU, T = 23.9 °C, and TOC = 3.5 mg·L^–1^. The pilot study essentially mimicked the reuse of highly treated wastewater effluent through the treatments at a DWTP. It is possible that some PFAA precursors had undergone transformation during the advanced treatment processes at the WWTP before entering the pilot plant; however, those precursors were beyond the consideration of this study. The ozone contactor at the pilot plant consisted of four 8 in. diameter, 20 ft tall ozone columns in series with main-stream ozone injection, and equipped with multiple sampling ports. The total volume of all four columns was approximately 180 gallons, which provided a total contact time of 40 min at a flow rate of 4.5 gpm.

The pilot testing for ozonation and ozone/H_2_O_2_ trials were conducted on 2 days. The pilot ozonation system was tested on the first day, and the initial applied O_3_ concentration was set at 1.0, 2.0, and 4.0 mg·L^–1^, respectively. On the next day, a variable speed peristaltic pump was added for dosing an aqueous hydrogen peroxide (H_2_O_2_) agent at the ozone contact time of 2.7 min for the pilot ozone/H_2_O_2_ system. The initial applied O_3_ concentration was set at 3.0 mg·L^–1^, and dose of H_2_O_2_ was set at 0, 0.05, and 0.20 mg·L^–1^, respectively (system with the initial dose of O_3_ = 3.0 mg·L^–1^/H_2_O_2_ = 0 mg·L^–1^ was also considered as the ozonation process in the Results and Discussion section). All water samples for PFAS analysis were collected at contact times of 0, 5, 10, and 20 min in 1 L high-density polyethylene (HDPE) bottles (prerinsed with methanol and DI water) and kept cold with ice during transportation for no more than 4 h. Upon return to the laboratory, all water samples were stored at 4 °C and extracted within 14 days. Water samples were also collected at contact times of 1.2, 5, 10, and 20 min in 100 mL glass beakers (prerinsed with methanol and DI water) and immediately analyzed for ozone residual concentration using the Hach Ozone Accuvac test kit. Online ozone analyzers were also used at sampling locations for residual ozone measurements.

### 2.3 TOP Assay

The TOP assay can estimate the levels of possible precursors in wastewater samples by transforming precursors into PFAAs for which analytical standards exist.^40−42^ Previous research has also demonstrated that PFAAs are not oxidized at an appreciable rate by the TOP assay. Thus, the TOP assay can measure the total concentration of unknown oxidizable PFAS that are not measurable by direct analysis. The TOP assay applies a heat-activated persulfate under alkaline conditions (pH > 12), which quickly generates sulfate (SO_4_^•–^) and hydroxyl (^•^OH) radicals that convert precursors to PFAAs without oxidizing PFAAs. Sample aliquots (125.0 mL) were transferred to prerinsed 250 mL HDPE bottles and added with 125.0 mL of 200.0 mM potassium persulfate and 12.5 mL of 10 M NaOH to yield pH > 12.0 [with minimum headspace (<20 mL) finally]. The mixtures were well agitated and sonicated for 5 min. Bottles were placed in a temperature-controlled water bath at 80–85 °C for more than 6 h. After cooling, mixtures were neutralized to be within pH 6.0–8.0 using 1.0 M HCl. Samples were stored for up to 1 day at 4 °C before extraction. Note that the control sample (DI water) was treated with the same procedures to check for any cross-contamination during the TOP assay.

### 2.4 Solid-Phase Extraction for Water Samples

All water samples and TOP samples were subjected to solid-phase extraction (SPE) with modified EPA methods (EPA 533, 537.1, and draft 1633).^44−46^ The SPE cartridges used were Strata-X-AW (33 μm Polymeric Weak Anion, 200 mg/6 mL) obtained from Phenomenex. PFASs were quantified using the relative response of analytes to their respective isotope-labeled surrogates (Supporting Information Table S1). Duplicate aliquots (250.0 mL each) of each sample were spiked with isotope-labeled surrogate mixtures (spiking concentration at 10.0 ng·L^–1^ before extraction). SPE cartridges were preconditioned on an SPE manifold with 10.0 mL of methanol, 10.0 mL of aqueous 0.1 M phosphate buffer, and 2.0 mL of DI water. Samples and rinsates from sample bottles (by 10.0 mL of 1.0 g·L^–1^ ammonium acetate followed by 1.0 mL of methanol) were both loaded into the cartridges at around 5 mL·min^–1^ (1–2 drops per second). Afterward, cartridges were rinsed with 5.0 mL of DI water and dried under a vacuum for 30 min. Dried cartridges were eluted with 10.0 mL of 2.0% ammonium hydroxide (v/v) in methanol into 15 mL polypropylene (PP) tubes. Extracts were evaporated to near dryness, reconstituted in 500 μL of 80/20 (v/v) methanol/H_2_O, vortexed, sonicated, and transferred to PP HPLC vials for storage at 4 °C until analysis by liquid chromatography time-of-flight mass spectrometry (LC-TOFMS).

### 2.5 LC-TOFMS Analysis

Extracted wastewater and TOP samples were injected on an Agilent 1260 Infinity HPLC instrument with 6230 TOFMS under electrospray ionization in the negative (ESI^–^) ion mode. Full scan mass spectra were acquired from 50 nm to 1000 m/z with a mass accuracy of within 10 ppm. The drying gas flow rate was 9 L min^–1^, gas temperature 200 °C, nebulizer pressure 40 psi, capillary voltage 4000 V, and fragmentation voltage 140, 180, 220, and 250 V. Mass accuracy was continually corrected by reference standards with reference masses of 119.036320 and 980.016375. Sample injections of 10.0 μL were separated using a Poroshell 120 EC–C18 column (2.1 × 150 mm, 2.7 μm). The chromatographic method consisted of a multistep gradient lasting 33 min at a constant 0.3 mL min^–1^ eluent flow rate with 5.0 mM ammonium acetate and 80/20 (v/v) methanol/acetonitrile. After 2 min hold at 100% of 5.0 mM ammonium acetate, the organic phase was ramped to 60% over 3 min, to 98% over 10 min, followed by a 7 min hold at 98%. The gradient was ramped to 100% of 5.0 mM ammonium acetate, followed by a 10 min postrun equilibration period. PFASs were quantified based on the ratio of the peak area of targeted PFAS to that of their respective isotope surrogates. The quality control (QC) of the MS analyses has been addressed previously,^43^ with new details related to this study provided in Supporting Information Text S2 and Table S2.

### 2.6 Statistical Analysis

Correlation analysis of PFAS concentrations with several operational/chemical descriptors (e.g., contact time and oxidant dose) was performed using the CORRELATION function in Microsoft Excel and linear fitting program in Origin 2021. Descriptors which exhibited correlation coefficients (absolute value) above 0.5 with p-value <0.05 were considered to have statistically significant correlation with different PFAS concentrations. In cases the descriptors have a strong correlation with PFAS concentrations, linear regression was performed.

## 3 Results and Discussion

### 3.1 PFAS Formation during Ozonation Processes

**Figure 1:**
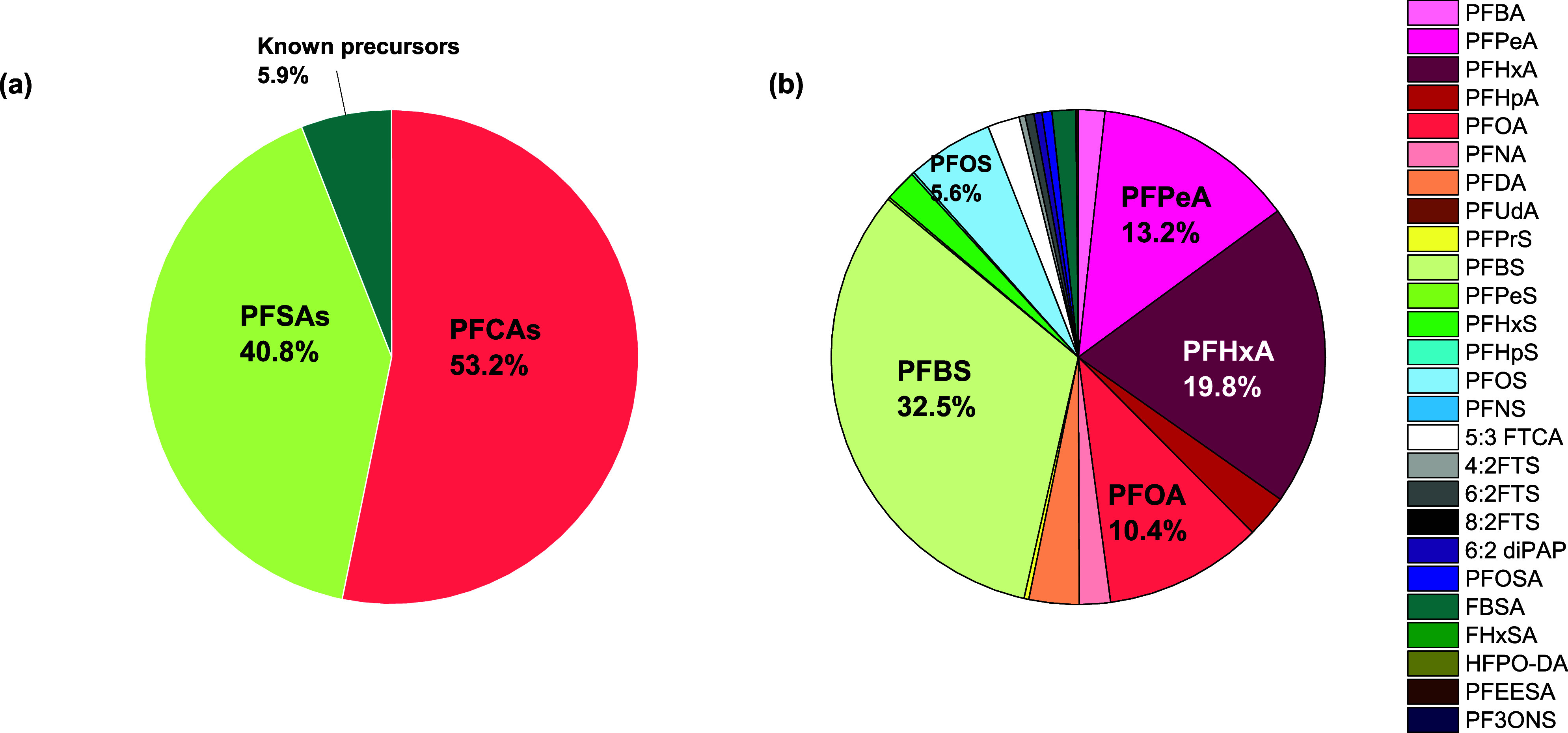
Concentration distributions (% of total) of (a) PFAS groups and (b) individual PFAS compounds in the influent wastewater entering the pilot plant in this study. PFAS concentration is based on ng·L^–1^.

**Figure 2:**
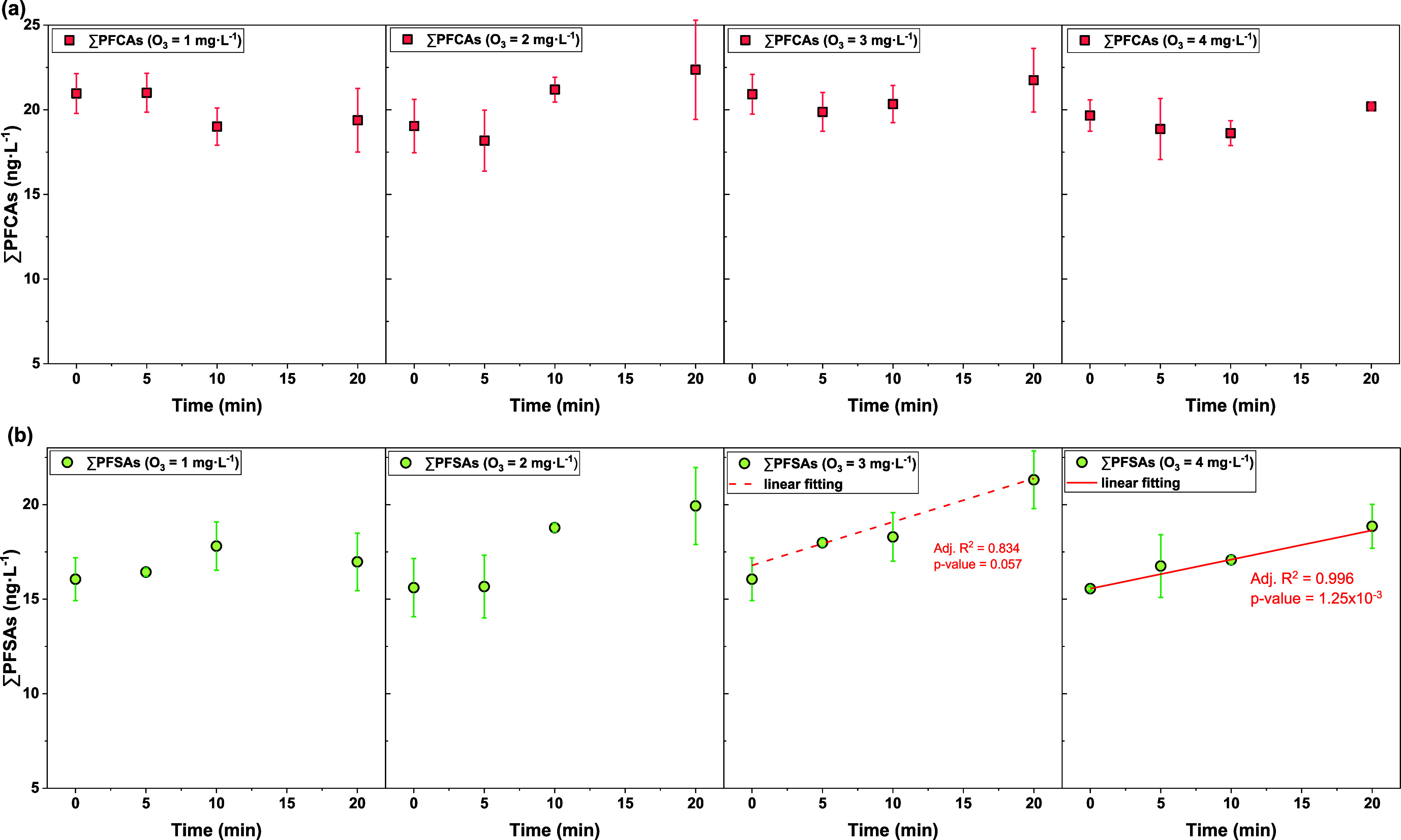
Averaged concentrations of classified PFAAs (ng·L^–1^) in ozone-treated water samples.

The results of ozone-treated samples collected from the pilot study are presented in Figure 1, 2, S2a, S3, and S5a and Tables S3, S5–S6. The concentration of residual ozone in water samples decreased with increasing contact time (Figure S2 and Table S3). 26 of the 40 monitored PFAS were detected in ozone-treated water samples, including PFBA, PFPeA, PFHxA, PFHpA, PFOA, PFNA, PFDA, PFUdA, PFPrS, PFBS, PFPeS, PFHxS, PFHpS, PFOS, PFNS, 5:3 FTCA, 4:2 FTS, 6:2 FTS, 8:2 FTS, 6:2 diPAP, PFOSA, FBSA, FHxSA, HFPO–DA, PFEESA, and 9Cl-PF3NOS. Figure 1 serves as an example to show the percent composition of individual PFAS compounds and PFAS classes (PFCA, PFSA, and known precursors) in the influent water sample before ozonation treatment at the pilot plant (O_3_ = 3.0 mg·L^–1^, time = 0 min). PFCAs and PFSAs were the dominant PFAS, while the known precursors only contributed to a small percentage of total PFAS in the influent water sample. After the ozonation process (measured at 20 min), the summed PFAA concentrations (∑_15_PFAA) changed from 37.0 to 36.4 ng·L^–1^ for O_3_ = 1.0 mg·L^–1^, from 34.6 to 42.3 ng·L^–1^ for O_3_ = 2.0 mg·L^–1^, from 37.0 to 43.1 ng·L^–1^ for O_3_ = 3.0 mg·L^–1^, and from 35.2 to 39.0 ng·L^–1^ for O_3_ = 4.0 mg·L^–1^, respectively. Total PFAS concentration, defined as ∑_15_PFAA and ∑_11_precursors, changed from 41.7 to 38.5 ng·L^–1^ for O_3_ = 1.0 mg·L^–1^, from 37.9 to 44.4 ng·L^–1^ for O_3_ = 2.0 mg·L^–1^, from 39.3 to 45.2 ng·L^–1^ for O_3_ = 3.0 mg·L^–1^, and from 38.2 to 41.4 ng·L^–1^ for O_3_ = 4.0 mg·L^–1^, respectively (Table S5 and S6).

#### 3.1.1 PFCAs and PFSAs

Of 11 PFCAs, eight PFCAs (C4–C11) were detected in all water samples treated by different ozone doses (1.0–4.0 mg·L^–1^) (Table S5 and S6). In all ozone-treated water samples, PFHxA (*C* = 6, 7.2, 9.0 ng·L^–1^) showed the highest concentration among the PFCAs detected, and average concentrations of PFCAs followed the trend: PFHxA (*C* = 6) > PFPeA (*C* = 5) > PFOA (*C* = 8) > PFHpA (*C* = 7) ≈ PFDA (*C* = 10) > PFBA (*C* = 4) > PFNA (*C* = 9) > PFUdA (*C* = 11). Figure 2a shows the overall concentrations of PFCAs over the contact time in water samples treated by various ozone dosages. After 20 min contact time with ozone, ∑_8_PFCA slightly decreased from 21.0 to 19.4 ng·L^–1^ for O_3_ = 1.0 mg·L^–1^; while increased from 19.0 to 22.4 ng·L^–1^ for O_3_ = 2.0 mg·L^–1^, from 20.9 to 21.7 ng·L^–1^ for O_3_ = 3.0 mg·L^–1^, and from 19.7 to 20.2 ng·L^–1^ for O_3_ = 4.0 mg·L^–1^, respectively. Except for water treated by a lower dose of ozone (O_3_ = 1.0 mg·L^–1^), ∑_8_PFCA increased during the ozonation treatment processes, with a 17.5% increase for O_3_ = 2.0 mg·L^–1^, a 4.0% increase for O_3_ = 3.0 mg·L^–1^, and a 2.7% increase for O_3_ = 4.0 mg·L^–1^, respectively. However, no obvious linear correlation between the contact time with ozone and PFCA concentrations during ozonation processes could be found. Several previous studies also reported that PFCA concentration would increase after the ozonation process.^39,47−50^ This increase in the concentrations of PFCAs may be due to the transformation of precursors present in water that could be oxidized by ozone. The ^•^OH generated from the decay of ozone could possibly play a role in these processes as well, but their contribution to transforming precursors should be much less than that to ozone, which will be further discussed in Section 3.2. For individual PFCA contribution to the elevated overall concentration, the highest increase was observed for PFPeA (*C* = 5), PFHxA (*C* = 6), and PFOA (*C* = 8), which were the dominant PFCAs detected in all water samples as well. Figure S3a–c shows the concentrations of these three PFCAs over contact time in water samples treated by various ozone dosages, respectively. Although analysis indicates that there is no statistically significant linear correlation between the ozone contact time and individual PFCA concentration, it is worth noting that the elevated PFOA concentration after ozonation (4.3 ng·L^–1^ treated by O_3_ = 2.0 mg·L^–1^, 4.4 ng·L^–1^ treated by O_3_ = 3.0 mg·L^–1^, respectively) exceeded the MCL in drinking water established by the USEPA (4 ng·L^–1^).^12^

As for PFSAs, seven PFSAs (C3–C9) were detected in all water samples treated by different ozone doses (1.0–4.0 mg·L^–1^) (Tables S5 and S6). PFBS was the predominant PFSA with concentrations ranging from 12.4 to 15.1 ng·L^–1^. Concentrations of PFSAs in ozone-treated water samples followed the trend of PFBS (*C* = 4) ≫ PFOS (*C* = 8) > PFHxS (*C* = 6) ≈ PFNS (*C* = 9) > PFPrS (*C* = 3) ≈ PFPeS (*C* = 5) ≈ PFHpS (*C* = 7). Figure 2b shows the overall concentrations of PFSAs over the contact time in water samples treated by various ozone dosages. After 20 min of contact time with ozone, ∑_7_PFSA (C3–C9) changed from 16.1 to 17.0 ng·L^–1^ for O_3_ = 1.0 mg·L^–1^, from 15.6 to 19.9 ng·L^–1^ for O_3_ = 2.0 mg·L^–1^, from 16.1 to 21.3 ng·L^–1^ for O_3_ = 3.0 mg·L^–1^, and from 15.6 to 18.9 ng·L^–1^ for O_3_ = 4.0 mg·L^–1^, respectively. ∑_7_PFSA increased during all ozonation treatment processes, with 5.7% increase for O_3_ = 1.0 mg·L^–1^, 27.7% increase for O_3_ = 2.0 mg·L^–1^, 32.8% increase for O_3_ = 3.0 mg·L^–1^, and 21.2% increase for O_3_ = 4.0 mg·L^–1^, respectively. Analysis also showed that the overall PFSA concentrations were linearly correlated with the contact time in water samples treated by higher initial ozone level at 3.0 mg·L^–1^ and showed statistically significant linear correlations (*p* < 0.05) at the initial ozone level of 4.0 mg·L^–1^ (Figure 2b and Table S9). These increasing trends indicated that longer contact time with ozone led to a higher concentration of PFSAs in water samples during ozonation processes. Several previous studies also reported that increased concentrations of some PFSAs (*C* < 8) were observed after ozonation treatment.^39,48^ The elevation of PFSA concentrations could be caused by the transformation of some precursors in water. Figure S3d,e shows the concentrations of two most abundant PFSAs, PFBS (*C* = 4) and PFOS (*C* = 8), over contact time in water samples treated by various ozone dosages. The increase in overall PFSAs was mainly attributed to the elevated PFBS concentration, with a 3.9% increase for O_3_ = 1.0 mg·L^–1^, 21.2% increase for O_3_ = 2.0 mg·L^–1^, 13.2% increase for O_3_ = 3.0 mg·L^–1^, and 15.2% increase for O_3_ = 4.0 mg·L^–1^, respectively. Analysis also indicated statistically significant linear correlations between the contact time with ozone and PFBS concentrations (*p* < 0.05) (Figure S3d and Table S9), that the longer contact time with ozone led to a higher concentration of PFBS in water samples during ozonation processes. Not only PFBS concentration was increased, there also existed the risk for PFOS concentration to exceed the USEPA MCL after ozonation (4.4 ng·L^–1^ after treated by O_3_ = 3.0 mg·L^–1^). No obvious linear correlation between the initial ozone dose and PFSA concentrations during ozonation processes could be found, but the observed increasing trend of the concentration of ∑_7_PFSA indicated that a higher dose of O_3_ (≥2.0 mg·L^–1^) could promote the formation of PFSAs. Furthermore, based on the results of this study (Tables S5 and S6), the maximum concentrations PFBA, PFHxS, PFNA, and GenX detected in all water samples were 15.1, 1.0, 0.8, and 0.2 ng·L^–1^, respectively. The risk of these PFAS mixtures violating the maximum HI of 1.0 was much lower compared with the risk of detected PFOA and PFOS exceeding the regulated MCL (4.0 ppt).

Overall, the ozonation treatment processes could have a moderate influence on increasing the concentration of PFAAs under practical conditions.

#### 3.1.2 Known PFAA-Precursors

Of the 21 known PFAA precursors, 11 precursors, 5:3 FTCA, 4:2 FTS, 6:2 FTS, 8:2 FTS, 6:2 diPAP, PFOSA, FBSA, FHxSA, HFPO–DA, PFEESA, and 9Cl-PF3NOS, were detected in ozone-treated water samples (Tables S5 and S6). Figure S5a shows the overall concentrations of the known precursors over contact time in water samples treated with various ozone dosages. After 20 min of contact time with ozone, ∑_11_Precursors changed from 4.7 to 2.2 ng·L^–1^ for O_3_ = 1.0 mg·L^–1^, from 3.2 to 2.1 ng·L^–1^ for O_3_ = 2.0 mg·L^–1^, from 2.3 to 2.2 ng·L^–1^ for O_3_ = 3.0 mg·L^–1^, and from 3.0 to 2.3 ng·L^–1^ for O_3_ = 4.0 mg·L^–1^, respectively. Among 11 detected precursors, 6:2 FTS was the most prevalent (2.3 ng·L^–1^) in influent water samples treated by low dose of ozone (O_3_ = 1.0 mg·L^–1^, *t* = 0 min), and its concentration dropped to 0.3 ng·L^–1^ after contacting with ozone for over 20 min. The change in 6:2 FTS concentration predominated the overall decrease in known precursors at O_3_ = 1.0 mg·L^–1^. For water samples treated by higher doses of ozone (O_3_ ≥ 2.0 mg·L^–1^), however, 6:2 FTS concentrations were much lower in the influent water samples (0.2–1.1 ng·L^–1^), which indicates that a higher dose of ozone or longer contact time with ozone may not lead to more efficient transformation of these precursors in water samples, as expected. In addition, although elevated concentrations of PFCAs and PFSAs were observed after ozonation as discussed in previous sections, based on the low initial concentrations and very limited decreases observed in known PFAA precursors (Δ_∑11precursors,max_ = 2.5 ng·L^–1^), it is difficult to link the transformation between specific decreased known precursor with specific increased PFAA. Therefore, the increased PFAAs after ozonation were more likely caused by the transformation of other unidentified precursors present in the influent water other than legacy precursor compounds, which will be further discussed in Section 3.3.

### 3.2 PFAS Formation during Ozone/H_2_O_2_ AOP

**Figure 3:**
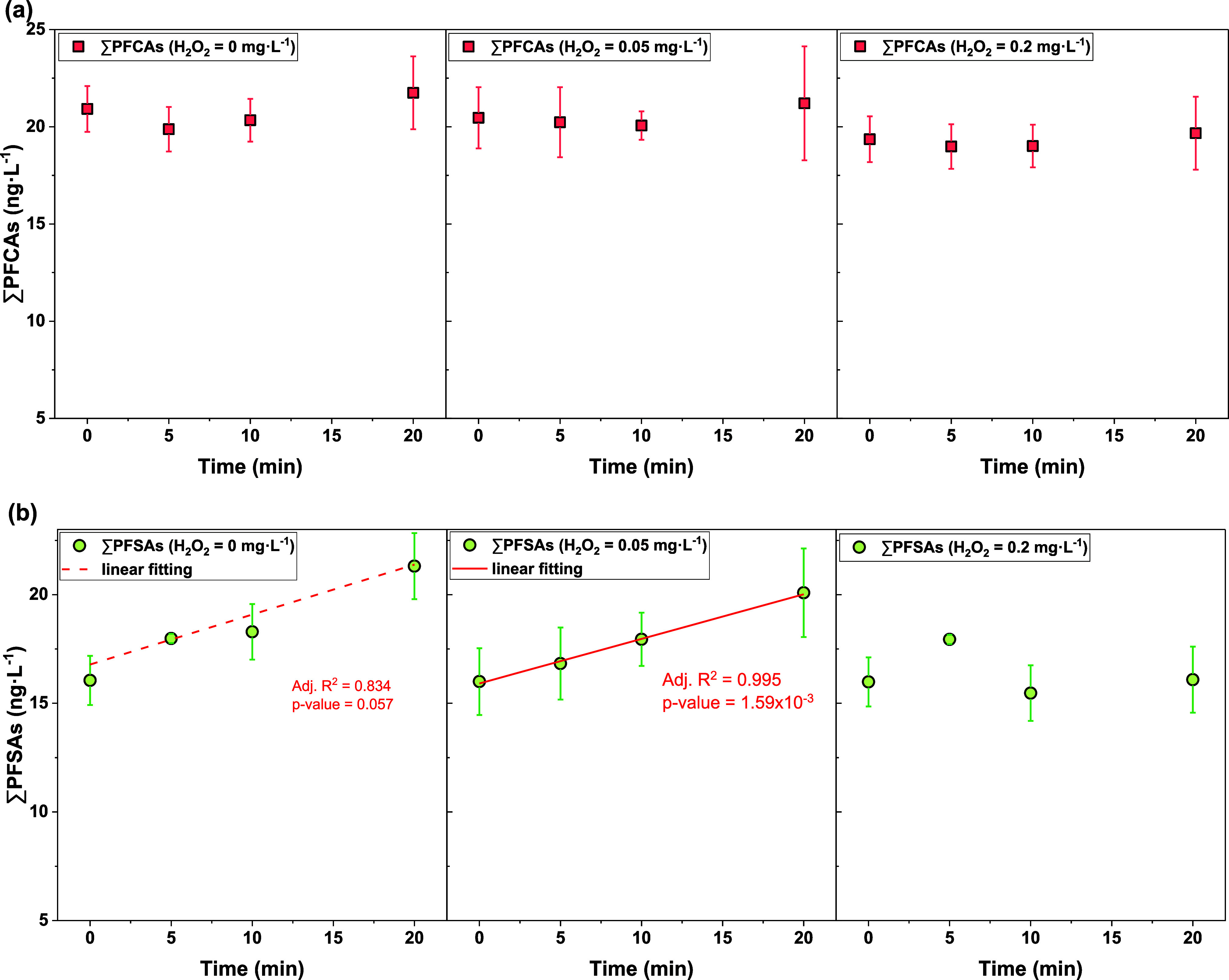
Averaged concentrations of classified PFAAs (ng·L^–1^) in ozone/H_2_O_2_ AOP-treated water samples.

**Figure 4:**
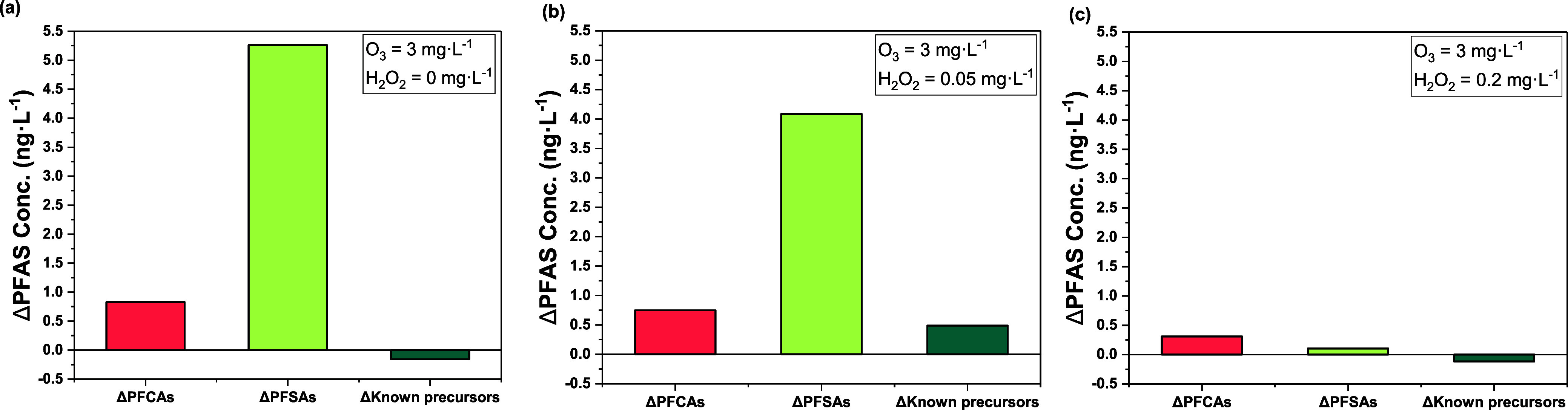
Overall change in PFAS concentrations (ng·L^–1^) in water samples treated by (a) ozonation and (b,c) ozone/H_2_O_2_ AOP processes; ΔPFAS = [PFAS]_*t*=20 min_ – [PFAS]_*t*=0 min_.

The results of ozone/H_2_O_2_ AOP-treated water samples collected from the pilot study are presented in Figures 3,4, S2b, S4, and S5b and Tables S4 and S6. Under the AOP conditions, the concentration of residual ozone in water samples dropped very quickly and significantly with the presence of H_2_O_2_ in the ozonation systems (Figure S2b and Table S4), presumably forming ^•^OH. 24 of the 40 monitored PFAS were detected in the water samples, including PFBA, PFPeA, PFHxA, PFHpA, PFOA, PFNA, PFDA, PFUdA, PFPrS, PFBS, PFPeS, PFHxS, PFHpS, PFOS, PFNS, 5:3 FTCA, 4:2 FTS, 6:2 FTS, 6:2 diPAP, PFOSA, FBSA, FHxSA, PFEESA, and 9Cl-PF3NOS. The summed PFAA concentrations (∑_15_PFAA) during ozone/H_2_O_2_ processes changed from 37.0 to 43.1 ng·L^–1^ for H_2_O_2_ = 0 mg·L^–1^, from 36.5 to 41.3 ng·L^–1^ for H_2_O_2_ = 0.05 mg·L^–1^, and from 35.3 to 35.8 ng·L^–1^ for H_2_O_2_ = 0.20 mg·L^–1^, respectively. Total PFAS concentration changed from 39.3 to 45.2 ng·L^–1^ for H_2_O_2_ = 0 mg·L^–1^, from 38.1 to 43.4 ng·L^–1^ for H_2_O_2_ = 0.05 mg·L^–1^, and from 37.5 to 37.8 ng·L^–1^ for H_2_O_2_ = 0.20 mg·L^–1^, respectively.

#### 3.2.1 PFCAs and PFSAs

Same as ozone-treated processes, eight PFCAs (C4–C11) were detected in ozone/H_2_O_2_-treated samples at different H_2_O_2_ doses (0–0.20 mg·L^–1^) with the same initial O_3_ dose of 3.0 mg·L^–1^ (Table S6). PFHxA (*C* = 6, 7.2–7.9 ng·L^–1^) still dominated among the PFCAs detected, and average concentrations of individual PFCAs followed the same trend as observed in ozone-treated water samples. Figure 3a shows the concentrations of PFCAs detected in ozone/H_2_O_2_ AOP-treated water samples. After 20 min of contact time with O_3_/H_2_O_2_, ∑_8_PFCA changed from 20.9 to 21.7 ng·L^–1^ for H_2_O_2_ = 0 mg·L^–1^, from 20.5 to 21.2 ng·L^–1^ for H_2_O_2_ = 0.05 mg·L^–1^, and from 19.4 to 19.7 ng·L^–1^ for H_2_O_2_ = 0.20 mg·L^–1^, respectively. The quick drop of residual ozone concentrations in water samples (Figure S2b and Table S4) indicated that the presence of hydrogen peroxide in the ozonation systems accelerated the decomposition of O_3_ into ^•^OH radicals. However, the formation of ^•^OH radicals did not significantly promote the formation of PFCAs in the ozone/H_2_O_2_ systems. Figure 4 shows the overall changes in the concentrations of PFAS classes (ΔPFCAs, ΔPFSAs, Δknown precursors) in water samples after treatment by ozonation and ozone/H_2_O_2_ AOP for 20 min. Compared to the increase in ∑_8_PFCA for water treated by ozone only (4.0% increase for the O_3_ = 3.0 mg·L^–1^/H_2_O_2_ = 0 mg·L^–1^), the ∑_8_PFCA only increased 3.7% for H_2_O_2_ = 0.05 mg·L^–1^ and 1.6% for H_2_O_2_ = 0.20 mg·L^–1^, respectively (Figure 4 and Table S6). For the change in the concentration of individual PFCA, the increase in H_2_O_2_ dosage did not promote the increase in PFOA concentrations either (Figure S4a and Table S6). There was no obvious linear correlation of the initial H_2_O_2_ dose or contact time with PFCA concentrations during the ozone/H_2_O_2_ processes. Since ^•^OH radicals have greater oxidation capability compared with those of O_3_, the limited elevation of concentration of PFCAs during ozone/H_2_O_2_ AOP might be attributed to the presence of natural organic matter (NOM) or other contaminants that could react with ^•^OH more effectively than PFAA precursors in water samples.

Seven PFSAs (C3–C9) were detected in all ozone/H_2_O_2_-treated water samples (Table S6). PFBS still showed the highest concentrations in PFSAs (12.4–14.5 ng·L^–1^). Figure 3b shows the overall concentrations of PFSAs over contact time in water samples treated with various H_2_O_2_ dosages. After 20 min of contact time with O_3_/H_2_O_2_, ∑_7_PFSA (C3–C9) changed from 16.1 to 21.3 ng·L^–1^ for H_2_O_2_ = 0 mg·L^–1^, from 16.0 to 20.1 ng·L^–1^ for H_2_O_2_ = 0.05 mg·L^–1^, and from 16.0 to 16.1 ng·L^–1^ for H_2_O_2_ = 0.20 mg·L^–1^, respectively (Table S6). Analysis also indicated statistically significant linear correlations between the contact time with ozone/H_2_O_2_ and PFBS concentrations (*p* < 0.05) at H_2_O_2_ = 0.05 mg·L^–1^ (Figure 3d and Table S9). However, although ∑_7_PFSA still increased during ozone/H_2_O_2_ AOP, a higher dose of H_2_O_2_ failed to promote the formation of PFSAs in water samples, with 32.8% increase for H_2_O_2_ = 0 mg·L^–1^, 25.6% increase for H_2_O_2_ = 0.05 mg·L^–1^, while only 0.69% increase for H_2_O_2_ = 0.2 mg·L^–1^, respectively (Figure 4 and Table S6). When looking into the concentrations of individual PFSAs, the drop of increased concentration of sum PFSAs was highly dependent on the limited formation of two dominated PFSAs, PFBS, and PFOS, with the presence of higher doses of H_2_O_2_ (Figure S4b,c). No obvious linear correlation between the initial H_2_O_2_ dose/contact time and PFSA concentrations during ozone/H_2_O_2_ AOP could be found. The limited transformation of PFSA precursors present in water samples, especially PFBS (*C* = 4) and PFOS (*C* = 8) precursors, could be attributed to the water matrix (e.g., NOM) scavenging or the infectiveness of ^•^OH radicals.

Overall, the ozone/H_2_O_2_ AOP treatment showed less potential on elevating the PFAA concentrations than ozonation processes under practical conditions.

#### 3.2.2 Known PFAA Precursors

Nine known precursors, 5:3 FTCA, 4:2 FTS, 6:2 FTS, 6:2 diPAP, PFOSA, FBSA, FHxSA, PFEESA, and 9Cl-PF3NOS, were detected in ozone/H_2_O_2_ AOP-treated water samples (Table S6). Figure S5b shows the overall concentrations of the known precursors over contact time in water samples treated with various H_2_O_2_ dosages. After 20 min of contact time with O_3_/H_2_O_2_, ∑_9_Precursors changed from 2.3 to 2.2 ng·L^–1^ (4.3% decrease) for H_2_O_2_ = 0 mg·L^–1^, from 1.6 to 2.1 ng·L^–1^ (31.2% increase) for H_2_O_2_ = 0.05 mg·L^–1^, and from 2.1 to 2.0 ng·L^–1^ (4.7% decrease) for H_2_O_2_ = 0.20 mg·L^–1^, respectively. Results suggested limited transformation of precursors in ozone/H_2_O_2_ AOP-treated water samples when a low dose of H_2_O_2_ was used, however, a higher dose of H_2_O_2_ could slightly promote the transformation of precursors. As previously stated, the limited transformation of precursors might be attributed to the scavenging effect of the real water matrix during ozone/H_2_O_2_ treatment processes.

### 3.3 Contribution of Unknown PFAA Precursors

**Figure 5:**
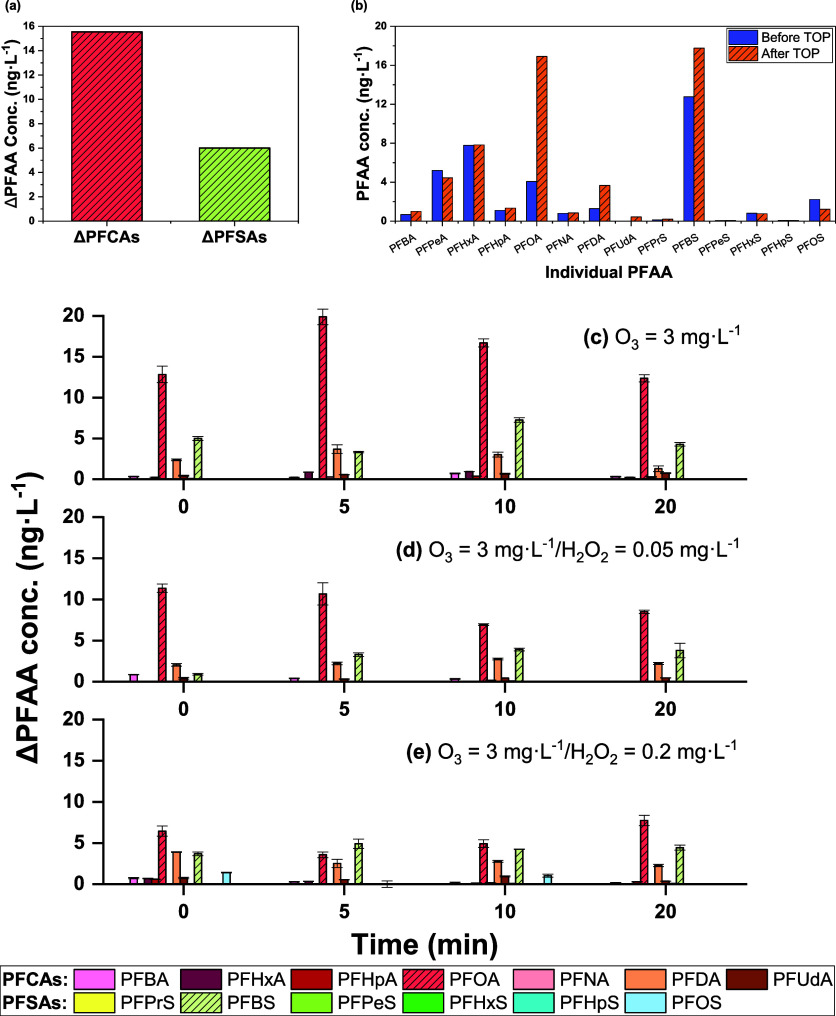
TOP assay results: (a) increase of classified PFAA concentrations (ΔPFAA, in ng·L^–1^) before and after TOP oxidation in the water samples without any ozonation (*t* = 0 min; ΔPFAA = [PFAA]_after TOP_ – [PFAA]_before TOP_); (b) concentrations of individual PFAA before and after TOP oxidation in the water samples without any ozonation (*t* = 0 min); (c–e) increase of PFAA concentrations (ΔPFAA, in ng·L^–1^) after TOP oxidation in water samples treated by various ozone/H_2_O_2_ doses.

The TOP assay was applied to approximate the contributions of the unknown PFAA precursors. Oxidation of each sample confirmed the presence of precursors that were not directly detected as specific target analytes. TOP oxidation converted precursors into a mixture of PFAAs, and the concentrations of total precursors can be estimated by comparing PFAA concentrations with corresponding chain lengths before and after TOP oxidation. Note that the TOP assay was applied to both ozonated and ozone/H_2_O_2_ AOP-treated water samples (O_3_ = 3.0 mg·L^–1^, H_2_O_2_ = 0–0.20 mg·L^–1^). Table S7 shows the measured concentrations of PFAS in wastewater samples after TOP oxidation. Figure 5 and Table S8 demonstrate the concentration changes in PFAAs compared with those before TOP oxidation.

As shown in Figure 5a, the concentrations of PFAAs significantly increased after TOP assay. Σ_8_PFCAs in ozone/AOP-treated water after TOP oxidation ranged from 25.6 to 44.5 ng·L^–1^ (before TOP: 19.0–21.7 ng·L^–1^) (Table S6, S7). The most significant increase was observed for long-chain PFCAs (*C* ≥ 8) and short-chain PFSAs (*C* < 6) (Δ_TOP_ of Σlong-chain PFCAs: 6.7–24.4 ng·L^–1^; Δ_TOP_ of Σshort-chain PFSAs: 1.0–7.3 ng·L^–1^, respectively) (Table S8), showing that precursors in water samples predominantly produced long-chain PFCAs and short-chain PFSAs. Among the transformation products, PFOA and PFBS were observed in the highest concentrations, suggesting the precursors for PFOA and PFBS predominated in the unknown precursors in the water samples. Figure 5b shows the concentrations of individual PFAA before and after TOP oxidation in the water samples without any treatment processes, which further supports the dominant presence of PFOA and PFBS precursors. The significant discrepancy between the increase in the PFAA concentration and the decrease in the known precursor concentration indicated that considerable amounts of unknown PFAA precursors were present in the wastewater, and their contribution to elevating PFAA concentrations during the oxidation treatment processes could not be ignored. Figure 5c-e shows the increase of PFAA concentrations after TOP oxidation for water samples treated by ozonation and ozone/H_2_O_2_ over the range of contact time. PFOA and PFBS still showed the highest increase during the treatment processes; however, the increase in PFAA concentrations became less significant as the H_2_O_2_ dose and contact time with H_2_O_2_ were increased, which indicated that the ozone/H_2_O_2_ AOP treatment had less potential in elevating the PFAA concentrations than ozonation processes.

The concentrations of 11 known precursors only slightly decreased after TOP oxidation compared to before TOP oxidation, with Σ_11_ known precursors in ozonated and ozone/H_2_O_2_-treated water after TOP oxidation ranging from 1.3 to 3.9 ng·L^–1^ (before TOP: 1.6–3.7 ng·L^–1^) (Tables S6 and S7). The limited transformation after TOP oxidation suggested that there could be incomplete oxidation, likely due to the presence of other coexisting contaminants and constituents in the water samples. It also indicated that the TOP assay conducted in this study might underestimate the concentration of unknown precursors in the wastewater samples and that the TOP assay may need to be optimized (e.g., alkaline conditions, temperature, and time) according to different source water samples in future studies.

## 4 Conclusions

Overall, this study showed the presence of eight PFCAs (C4–C11), seven PFSAs (C3–C9), and 11 precursors (5:3 FTCA, 4:2 FTS, 6:2 FTS, 8:2 FTS, 6:2 diPAP, PFOSA, FBSA, FHxSA, HFPO–DA, PFEESA, and 9Cl-PF3NOS) and their fate through two different oxidation treatment processes, ozonation and ozone/H_2_O_2_ AOP, in a pilot-scale wastewater reuse treatment plant. Short-chain (C3–C7) PFAAs are the dominant PFAS in all water samples. Additionally, the TOP assay revealed the presence of significant amounts of unknown PFAA precursors in the wastewater, where PFOA and PFBS precursors were in dominant concentrations. This study found that the concentrations of PFAAs could be moderately elevated throughout both ozonation and ozone/H_2_O_2_ AOP treatment processes under conditions mimicking real reuse applications of wastewater. Ozonation processes showed a more significant elevating effect on the concentration of PFAAs in water compared to ozone/H_2_O_2_ AOP. These results implied that ozone has a stronger impact on transforming PFAA precursors while ozone/H_2_O_2_ AOP is less effective possibly due to radical scavenging of real water matrices. Nevertheless, more research with different water sources is warranted to fully assess the impacts of ozonation versus ozone/H_2_O_2_ AOP on the PFAS transformation. Broadly, results of this study also have implications on direct or indirect portable reuse applications involving AOPs (e.g., ozone-based processes) treating less-pristine source waters. Future research should strengthen the monitoring and evaluation of the effect of ozone-based processes on the fate of PFAS and precursors in water treatment systems, particularly those with PFAS-impacted source waters.

## Supporting Information Available

Chemicals and reagents; analytical QC; PFAS analytes, retention time, and isotope-labeled surrogates; quality control sample results for PFAS analytes; residual ozone concentrations (mg·L^–1^) in water treated by ozonation and ozone/H_2_O_2_; PFAS concentrations (ng·L^–1^) in water samples treated by ozonation and ozone/H_2_O_2_; PFAS concentrations (ng·L^–1^) and increase of PFAA concentrations (ΔPFAA, in ng·L^–1^) after TOP oxidation in water samples treated by various ozone/H_2_O_2_ doses; statistical analysis between contact time (*t* in min) and classified PFAS concentrations (*c*_PFAS_ in ng·L^–1^) in water samples treated by ozonation and ozone/H_2_O_2_; pilot plant for testing and schematic diagrams of the treatment processes at pilot plant; residual ozone concentrations (mg·L^–1^) in water samples treated by ozonation and ozone/H_2_O_2_; averaged concentrations of representative individual PFAA (ng·L^–1^) in ozone-treated and ozone/H_2_O_2_ AOP-treated water samples; and averaged concentrations of known PFAS precursors (ng·L^–1^) in ozone-treated and ozone/H_2_O_2_ AOP-treated water samples (PDF)
