# Supplementary material for: Pilot Assessment of Impacts of Ozone and Ozone/Hydrogen Peroxide Treatment on the Fate of Per- and Polyfluoroalkyl Substances and Precursors
Source: ACS ES T Water. 2024 Sep 25;4(10):4545–55. doi: 10.1021/acsestwater.4c00565 (PMC11474954; doi:10.1021/acsestwater.4c00565)
Supplement: Supplementary file 1 — ew4c00565_si_001.pdf [file ew4c00565_si_001.pdf]

Supporting Information:

Pilot Assessment of Impacts of Ozone and Ozone/Hydrogen Peroxide Treatment on the Fate of Per- and Polyfluoroalkyl Substances and Precursors

Xiaoyue Xin,[a] Juhee Kim,[b] ShihChi Weng,[c] and Ching-Hua Huang[a,*]

[a]School of Civil and Environmental Engineering, Georgia Institute of Technology, Atlanta, GA 30332, United States

[b]Department of Civil, Environmental and Construction Engineering, University of Hawai`i at Mānoa, Honolulu, HI 96822, United States

[c]Department of Water Resources, Gwinnet County Government, Lawrenceville, GA 30045, United States

[*]Corresponding Authors. Emails: ching-hua.huang@ce.gatech.edu (Ching-Hua Huang)

Number of Pages: 22

Number of Texts: 2

Number of Tables: 9

Number of Figures: 5

Contents

Texts

Text S1. Chemicals and reagents. --S3

Text S2. Analytical quality control (QC). --S5

Tables

Table S1. PFAS analytes, retention time, and isotope-labeled surrogates.---------------------------S6

Table S2. Quality control sample results for PFAS analytes.---S7

Table S3. Residual ozone concentrations ($mg\cdot L^{-1}$) in water treated by various ozone doses.----- S8

Table S4. Residual ozone concentrations ($mg\cdot L^{-1}$) in water treated by various H_2O_2 doses.------ S8

Table S5. PFAS concentrations ($ng\cdot L^{-1}$) in water samples treated by various ozone doses.------ S9

Table S6. PFAS concentrations ($ng\cdot L^{-1}$) in water samples treated by various ozone/H_2O_2 doses.--- --- S11

Table S7. PFAS concentrations ($ng\cdot L^{-1}$) after TOP oxidation in water samples treated by various ozone/H_2O_2 doses.--- S14

Table S8. The increase of PFAA concentrations (ΔPFAA, in $ng\cdot L^{-1}$) after TOP oxidation in water samples treated by various ozone/H_2O_2 doses.--- S15

Table S9. Statistical analysis between contact time (*t* in min) and classified PFAS concentrations (c_{PFAS} in $ng\cdot L^{-1}$) in water samples treated by ozonation and ozone/H_2O_2.-------------- S16

Figures

Figure S1. (a) Photo of pilot plant for testing. (b) Schematic diagrams of the treatment processes at pilot plant.--- S17

Figure S2. Residual ozone concentrations ($mg\cdot L^{-1}$) in (a) water samples treated by various ozone doses; (b) water samples treated by various H_2O_2 doses (initial O_3 dose = 3 $mg\cdot L^{-1}$).- S18

Figure S3. Averaged concentrations of representative individual PFAA ($ng\cdot L^{-1}$) in ozone-treated water samples: (a) PFPeA; (b) PFHxA; (c) PFOA; (d) PFBS; (e) PFOS.--------------- S19

Figure S4. Averaged concentrations of representative individual PFAA ($ng\cdot L^{-1}$) in ozone/H_2O_2 AOP-treated water samples: (a) PFOA; (b) PFBS; (c) PFOS.---------------------------- S21

Figure S5. Averaged concentrations of known PFAS precursors ($ng\cdot L^{-1}$) in ozone-treated and ozone/H_2O_2 AOP-treated water samples.--- S22

Supplemental Methods

Text S1. Chemicals and reagents

Sodium phosphate dibasic (≥99%; MilliporeSigma), sodium phosphate monobasic (≥99.0%; MilliporeSigma), sodium hydroxide (97%; Thermo Fisher Scientific), ammonium hydroxide (30% NH_3 basis, MilliporeSigma), ammonium acetate (7.5 M solution; MilliporeSigma), and acetic acid (≥99%; MilliporeSigma) were used as solid-phase extraction (SPE) reagents. LC-MS grade methanol (Fisher Optima), LC-MS grade acetonitrile (Fisher Optima), acetic acid (Fisher Optima), LC-MS grade ammonium acetate (Fisher Optima), and Milli-Q water were used to prepare LC eluent. All 40 PFAS were purchased from Wellington Laboratories (Guelph, Canada), including: perfluorobutanoic acid (PFBA), perfluoropentanoic acid (PFPeA), perfluorohexanoic acid (PFHxA), perfluoroheptanoic acid (PFHpA), perfluorooctanoic acid (PFOA), perfluorononanoic acid (PFNA), perfluorodecanoic acid (PFDA), perfluoroundecanoic acid (PFUnA), perfluorododecanoic acid (PFDoA), perfluorortridecanoic acid (PFTrDA), perfluorotetradecanoic acid (PFTeDA), perfluoropropane sulfonic acid (PFPrS), perfluorobutane sulfonic acid (PFBS), perfluoropentane sulfonic acid (PFPS), perfluorohexane sulfonic acid (PFHxS), perfluorheptane sulfonic acid (PFHpS), perfluorooctane sulfonic acid (PFOS), perfluorononane sulfonic acid (PFNS), perfluorodecane sulfonic acid (PFDS), 4:2 Fluorotelomer sulfonic acid (4:2 FTS), 6:2 fluorotelomer sulfonic acid (6:2 FTS), 8:2 fluorotelomer sulfonic acid (8:2 FTS), 6:2 fluorotelomer carboxylic acid (6:2 FTCA), 5:3 fluorotelomer carboxylic acid (5:3 FTCA), 6:2 fluorotelomer unsaturated carboxylic acid (6:2 FTUCA), perfluorobutane sulfonamide (FBSA), perfluorohexane sulfonamide (FHxSA), perfluorooctane sulfonamide (PFOSA), N-methyl-perfluoro-1-octanesulfonamidoacetic acid (N-MeFOSAA), N-ethyl-perfluoro-1-octanesulfonamidoacetic acid (N-EtFOSAA), hexafluoropropylene oxide-dimer acid (HFPO-DA), 4,8-dioxa-3H-perfluorononanoic acid

(ADONA), 9-chlorohexadecafluoro-3-oxanone-1-sulfonic acid (9Cl-PF3ONS), 11-chloroeicosafluoro-3-oxaundecane-1-sulfonic acid (11Cl-PF3OUdS), perfluoro(2-ethoxyethane)sulfonic acid (PFEESA), perfluoro-3-methoxypropanoic acid (PF4OPeA), perfluoro-4-methoxybutanoic acid (PF5OHxA), nonafluoro-3,6-dioxaheptanoic acid (3,6-OPFHpA), 6:2 fluorotelomer phosphate diester (6:2 diPAP), and 6:2/8:2 fluorotelomer phosphate diester (6:2/8:2 diPAP).

Twenty-three isotope-labeled surrogates were purchased from Wellington Laboratories (Guelph, ON): [$^{13}C_4$] PFBA, [$^{13}C_5$] PFPeA, [$^{13}C_5$] PFHxA, [$^{13}C_4$] PFHpA, [$^{13}C_8$] PFOA, [$^{13}C_9$] PFNA, [$^{13}C_6$] PFDA, [$^{13}C_7$] PFUnA, [$^{13}C_2$] PFDoA, [$^{13}C_2$] PFTeA, [$^{13}C_3$] PFBS, [$^{13}C_5$] PFHxS, [$^{13}C_8$] PFOS, [$^{13}C_2$] 4:2 FTS, [$^{13}C_2$] 6:2 FTS, [$^{13}C_2$] 8:2 FTS, [$^{13}C_2$] 6:2 FTCA, [$^{13}C_2$] 6:2 FTUCA, [$^{13}C_8$] PFOSA, [$^{2}D_3$] N-MeFOSAA, [$^{2}D_5$] N-EtFOSAA, [$^{13}C_3$] HFPO-DA, and [$^{13}C_2$] 6:2 diPAP.

Text S2. Analytical quality control (QC).

To account for the matrix effects and extraction recovery, 23 isotope-labeled surrogates were used as internal standards. A 10-11 point calibration curve ranging from 0.06 to 10 $\mu g \cdot L^{-1}$ was conducted to cover the entire range of the sample concentrations at the beginning. A continuing calibration check was also conducted at every 10 injections and at the end of the analytical sequence to check the calibration. An instrument blank consisting of 80/20 (v/v) MeOH/H_2O was analyzed after the injection of the highest calibration standard to check the potential carryover between injections. The QC samples (extraction blanks and spiked DI water) were prepared to ensure accurate and reproducible PFAS measurements and to check the background contamination during the extraction procedure across batches. Results were present in Table S2. In extraction of blank samples (DI water; n = 6), 16 of 40 PFAS were detected but at very low concentrations of 0.06-0.33 $ng \cdot L^{-1}$. DI water spiked with 10.0 $ng \cdot L^{-1}$ of PFAS (n = 7) yielded recoveries of 75.4%–137.4% except for FBSA (33.8%). The measurements for replicate DI water spikes were precise, with percent relative standard deviations range within 1.3%–11.2% for individual analyzed PFAS, and 1.5% for summed PFCA class, 1.8% for summed PFSA class, and 3.2% for summed known precursors, respectively (see Table S2 for details). Method detection limits (MDLs), also treated as the limit of detection (LOD), were 0.06 $ng \cdot L^{-1}$ for all analytes, except for PFHpA and 4:2 FTS (0.08 $ng \cdot L^{-1}$). Values greater than or equal to the LOD were quantitated, and values below the LOD were reported as "<LOD". Field blank samples consisting of laboratory grade water poured into the sample bottle were collected alongside all treated water samples. The concentrations of PFAS detected in field blanks were less than LOD values and therefore no blank subtraction was employed on water samples. These replicate measurements demonstrated reproducibility and consistency in the analytical method.

Table S1. PFAS analytes, retention time, and isotope-labeled surrogates.

No.	Analyte	Theoretical m/z	Retention Time (min)	Isotope-labeled Surrogate	Theoretical m/z
1	PFBA	212.9792	7.6	[$^{13}C_4$] PFBA	216.9926
2	PFPeA	262.9760	8.6	[$^{13}C_5$] PFPeA	267.9928
3	PFHxA	312.9728	9.4	[$^{13}C_5$] PFHxA	317.9896
4	PFHpA	362.9696	10.2	[$^{13}C_4$] PFHpA	366.9830
5	PFOA	412.9664	11.1	[$^{13}C_8$] PFOA	420.9933
6	PFNA	462.9632	12.1	[$^{13}C_9$] PFNA	471.9934
7	PFDA	512.9600	13.0	[$^{13}C_6$] PFDA	518.9802
8	PFUnA	562.9568	13.8	[$^{13}C_7$] PFUnA	569.9803
9	PFDoA	612.9537	14.5	[$^{13}C_2$] PFDoA	614.9604
10	PFTrDA	662.9505	15.2	[$^{13}C_2$] PFTeA	714.9540
11	PFTeDA	712.9473	15.7	[$^{13}C_2$] PFTeA	714.9540
12	PFPrS	248.9462	8.2	[$^{13}C_3$] PFBS	301.9531
13	PFBS	298.9430	8.9	[$^{13}C_3$] PFBS	301.9531
14	PFPeS	348.9398	9.6	[$^{13}C_5$] PFHxS	401.9467
15	PFHxS	398.9366	10.4	[$^{13}C_3$] PFHxS	401.9467
16	PFHpS	448.9334	11.3	[$^{13}C_8$] PFOS	506.9571
17	PFOS	498.9302	12.3	[$^{13}C_8$] PFOS	506.9571
18	PFNS	548.9270	13.1	[$^{13}C_6$] PFDA	518.9802
19	PFDS	598.9238	13.9	[$^{13}C_7$] PFUnA	569.9803
20	4:2 FTS	326.9743	9.3	[$^{13}C_2$] 4:2 FTS	328.9810
21	6:2 FTS	426.9679	11.0	[$^{13}C_2$] 6:2 FTS	428.9746
22	8:2 FTS	526.9679	12.9	[$^{13}C_2$] 8:2 FTS	528.9682
23	6:2FTCA	376.9853	10.3	[$^{13}C_2$] 6:2 FTCA	378.9920
24	5:3FTCA	341.0041	10.2	[$^{13}C_2$] 6:2 FTCA	378.9920
25	6:2 FTUCA	356.9790	10.3	[$^{13}C_2$] 6:2 FTUCA	358.9858
26	FBSA	297.9590	9.7	[$^{13}C_3$] PFHxS	401.9467
27	FHxSA	397.9526	11.7	[$^{13}C_8$] PFOS	506.9571
28	PFOSA	497.9462	13.8	[$^{13}C_8$] PFOSA	505.9730
29	N-MeFOSAA	569.9673	13.3	[$^{2}D_3$] N-MeFOSAA	572.9862
30	N-EtFOSAA	583.9830	13.8	[$^{2}D_5$] N-EtFOSAA	589.0144
31	HFPO-DA	284.9779	9.6	[$^{13}C_3$] HFPO-DA	286.9846
32	ADONA	376.9689	10.4	[$^{13}C_3$] PFHpA	366.9830
33	9Cl-PF3ONS	530.8956	12.8	[$^{13}C_8$] PFOS	506.9571
34	11Cl-PF3OUdS	630.8892	14.3	[$^{13}C_7$] PFUnA	569.9803
35	PFEESA	314.9379	9.2	[$^{13}C_3$] PFBS	301.9531
36	PF4OPeA	228.9741	8.1	[$^{13}C_4$] PFBA	216.9926
37	PF5OHxA	278.9709	8.9	[$^{13}C_5$] PFPeA	267.9928
38	3,6-OPFHpA	200.9792	9.3	[$^{13}C_5$] PFHxA	317.9896
39	6:2 diPAP	788.9751	15.5	[$^{13}C_2$] 6:2 diPAP	792.9885
40	6:2/8:2 diPAP	888.9687	16.3	[$^{13}C_2$] 6:2 diPAP	792.9885

Table S2. Quality control sample results for PFAS analytes.

No.	Analyte	Extraction Blanks (n=6)		DI Water Spikes (10.0 ng/L, n=7)		LOD[c]
		n Detects	Max(ng·L^{-1})	Mean %R[a]	%RSD[b]	(ng·L^{-1})
1	PFBA	6	0.06	103.7	3.0	0.06
2	PFPeA	4	0.08	97.4	4.2	0.06
3	PFHxA	6	0.06	92.8	6.6	0.06
4	PFHpA	3	0.12	96.5	2.9	0.08
5	PFOA	6	0.29	104.4	5.3	0.06
6	PFNA	4	0.14	94.8	1.3	0.06
7	PFDA	3	0.12	102.5	3.7	0.06
8	PFUdA	0	0	96.3	5.3	0.06
9	PFDoA	0	0	88.6	6.2	0.06
10	PFTrDA	0	0	83.9	5.4	0.06
11	PFTeDA	0	0	90.2	4.4	0.06
*	PFCAs	/	/	/	**1.5**[d]	/
12	PFPrS	3	0.12	82.5 (n=5)	6.2	0.06
13	PFBS	6	0.22	97.8	3.0	0.06
14	PFPeS	0	0	96.2	3.0	0.06
15	PFHxS	0	0	96.9	2.1	0.06
16	PFHpS	0	0	106.7	6.4	0.06
17	PFOS	6	0.09	101.5	5.2	0.06
18	PFNS	0	0	83.3	5.3	0.06
19	PFDS	0	0	93.1	7.0	0.06
*	PFSAs	/	/	/	**1.8**	/
20	6:2 FTCA	0	0	124.3 (n=5)	6.8	0.06
21	6:2 FTCUA	0	0	131.3 (n=5)	7.7	0.06
22	5:3 FTCA	3	0.30	122.2 (n=5)	9.2	0.06
23	4:2 FTS	4	0.06	97.6 (40 ng/L)	3.5	0.08
24	6:2 FTS	6	0.33	137.4 (40 ng/L)	11.2	0.06
25	8:2 FTS	0	0	99.5 (40 ng/L)	2.3	0.06
26	6:2 diPAP	1	0.06	119.9 (n=5)	6.6	0.06
27	6:2/8:2 diPAP	0	0	126.6 (n=5)	8.4	0.06
28	PFOSA	2	0.06	102.8	5.5	0.06
29	NMeFOSAA	0	0	95.8	9.9	0.06
30	NEtFOSAA	0	0	98.0	4.8	0.06
31	FBSA	0	0	33.8	5.5	0.06
32	FHxSA	1	0.06	75.4	9.9	0.06
33	9Cl-PF3ONS	0	0	87.5	3.6	0.06
34	11Cl-PF3OUdS	0	0	84.0	7.5	0.06
35	HFPO-DA	0	0	96.6	2.4	0.06
36	ADONA	0	0	94.3	2.3	0.06
37	PFMPA	0	0	89.9	3.8	0.06
38	PFMBA	0	0	103.0	8.4	0.06
39	PFEESA	0	0	92.7	3.7	0.06
40	NFDHA	0	0	98.1	3.0	0.06
*	Known precursors	/	/	/	**3.2**	/

[a] %R = percent recovery.
[b] %RSD = percent relative standard deviation.
[c] LOD = limit of detection based on three times the signal to noise ratio.
[d] %RSD of summed concentrations of PFAS classes (PFCA, PFSA and known precursors).

Table S3. Residual ozone concentrations ($mg{\cdot}L^{-1}$) in water treated by various ozone dose.

Time (min)	Residual O_3 $mg{\cdot}L^{-1}$	O_3 Dose 1.0 $mg{\cdot}L^{-1}$	2.0 $mg{\cdot}L^{-1}$	3.0 $mg{\cdot}L^{-1}$	4.0 $mg{\cdot}L^{-1}$
1.2	**Average**[a]	**0.47**	**1.71**[b]	**1.43**	**0.95**
	STDEV	0.00	0.04	0.05	0.03
5	**Average**	**0.33**	**1.59**[b]	**1.17**	**0.79**
	STDEV	0.04	0.15	0.08	0.04
10	**Average**	**0.18**	**1.48**	**1.04**	**0.58**
	STDEV	0.04	0.20	0.08	0.07
20	**Average**	**0.07**	**1.13**	**0.64**	**0.36**
	STDEV	0.01	0.05	0.09	0.02

[a] Average value and standard deviation for the duplicate.

[b] Exceed the detection range (0 - 1.50 $mg{\cdot}L^{-1}$ O_3) of Ozone Accuvac® test kit.

Table S4. Residual ozone concentrations ($mg{\cdot}L^{-1}$) in water treated by various H_2O_2 dose (initial O_3 dose = 3.0 $mg{\cdot}L^{-1}$).

Time (min)	Residual O_3 $mg{\cdot}L^{-1}$	H_2O_2 Dose 0 $mg{\cdot}L^{-1}$	0.05 $mg{\cdot}L^{-1}$	0.20 $mg{\cdot}L^{-1}$
1.2	**Average**[a]	**1.43**	**1.35**	**1.20**
	STDEV	0.05	0.09	0.15
5	**Average**	**1.17**	**0.30**	**0.00**
	STDEV	0.08	0.09	0.00
10	**Average**	**1.04**	**0.04**	**0.00**
	STDEV	0.08	0.04	0.00
20	**Average**	**0.64**	**0.00**	**0.00**
	STDEV	0.09	0.00	0.00

[a] Average value and standard deviation for the duplicate.

Table S5. PFAS concentrations (ng·L^{-1}) in water samples treated by various ozone doses.

PFAS	O_3 dose	1.0 mg·L^{-1}				2.0 mg·L^{-1}				4.0 mg·L^{-1}			
	Time (min)	0	5	10	20	0	5	10	20	0	5	10	20
PFBA	**Average[a]**	**0.7**	**0.7**	**0.7**	**0.7**	**0.6**	**0.6**	**0.7**	**0.8**	**0.7**	**0.7**	**0.7**	**0.7**
	STDEV[a]	0.0	0.0	0.1	0.1	0.2	0.1	0.0	0.0	0.1	0.1	0.1	0.1
PFPeA	**Average**	**5.3**	**4.9**	**4.6**	**4.6**	**4.4**	**4.2**	**5.0**	**5.2**	**4.7**	**4.5**	**4.5**	**5.0**
	STDEV	0.4	0.2	0.7	0.7	0.6	0.3	0.4	0.4	0.4	1.0	0.2	0.4
PFHxA	**Average**	**7.9**	**9.0**	**7.6**	**7.5**	**7.2**	**7.7**	**9.0**	**8.9**	**7.6**	**7.7**	**7.7**	**7.8**
	STDEV	0.8	1.8	0.1	0.1	0.3	0.5	1.0	2.0	0.2	0.5	0.3	0.6
PFHpA	**Average**	**1.3**	**1.1**	**0.9**	**1.0**	**1.0**	**0.8**	**1.1**	**1.1**	**1.0**	**1.0**	**0.9**	**1.0**
	STDEV	0.5	0.1	0.1	0.0	0.0	0.1	0.1	0.0	0.2	0.2	0.1	0.0
PFOA	**Average**	**3.8**	**3.5**	**3.7**	**3.7**	**3.7**	**3.3**	**3.6**	**4.3**	**3.7**	**3.3**	**3.4**	**3.6**
	STDEV	0.4	0.5	0.1	0.7	0.5	0.4	0.1	0.1	0.2	0.5	0.3	0.2
PFNA	**Average**	**0.8**	**0.7**	**0.6**	**0.8**	**0.8**	**0.8**	**0.8**	**0.8**	**0.6**	**0.6**	**0.6**	**0.7**
	STDEV	0.0	0.1	0.0	0.0	0.2	0.0	0.0	0.1	0.1	0.1	0.0	0.1
PFDA	**Average**	**1.1**	**1.0**	**0.8**	**1.1**	**1.3**	**0.7**	**0.8**	**1.2**	**1.3**	**1.0**	**0.8**	**1.2**
	STDEV	0.0	0.1	0.1	0.2	0.7	0.2	0.0	0.3	0.1	0.4	0.2	0.3
PFUdA	**Average**	**<LOD[b]**	**<LOD**	**<LOD**	**<LOD**	**<LOD**	**<LOD**	**0.1**	**0.1**	**0.1**	**0.1**	**0.1**	**0.1**
	STDEV							0.0	0.1	0.0	0.0	0.0	0.0
PFPrS	**Average**	**0.2**	**0.2**	**0.2**	**0.2**	**0.2**	**0.1**	**0.3**	**0.2**	**0.1**	**0.1**	**0.2**	**0.2**
	STDEV	0.1	0.1	0.0	0.0	0.2	0.1	0.1	0.0	0.0	0.0	0.0	0.0
PFBS	**Average**	**13.0**	**13.1**	**14.2**	**13.5**	**12.5**	**12.4**	**14.9**	**15.1**	**12.4**	**13.2**	**13.5**	**14.3**
	STDEV	0.8	0.2	1.3	2.4	1.0	1.3	1.6	2.0	0.0	1.4	0.0	1.1
PFPeS	**Average**	**0.1**	**0.1**	**0.1**	**0.1**	**0.1**	**0.1**	**0.1**	**0.1**	**0.1**	**0.1**	**0.1**	**0.1**
	STDEV	0.0	0.0	0.0	0.0	0.0	0.0	0.0	0.0	0.0	0.0	0.0	0.0
PFHxS	**Average**	**0.8**	**0.7**	**0.8**	**0.8**	**0.8**	**0.7**	**0.8**	**1.0**	**0.8**	**0.7**	**0.7**	**0.8**
	STDEV	0.1	0.0	0.1	0.0	0.0	0.0	0.0	0.1	0.1	0.1	0.0	0.1
PFHpS	**Average**	**0.1**	**0.2**	**0.2**	**0.2**	**0.1**	**0.3**	**0.4**	**0.4**	**0.1**	**0.4**	**0.4**	**0.4**
	STDEV	0.0	0.0	0.1	0.0	0.0	0.0	0.0	0.1	0.0	0.1	0.1	0.1
PFOS	**Average**	**1.9**	**1.8**	**2.0**	**1.9**	**1.9**	**1.6**	**1.5**	**2.2**	**2.0**	**1.6**	**1.6**	**2.3**
	STDEV	0.2	0.1	0.7	0.2	0.3	0.0	0.1	0.5	0.0	0.2	0.2	0.0
PFNS	**Average**	**<LOD**	**0.4**	**0.4**	**0.3**	**<LOD**	**0.4**	**0.8**	**0.9**	**0.1**	**0.7**	**0.7**	**0.8**
	STDEV		0.1	0.1	0.1		0.0	0.0	0.3	0.0	0.0	0.0	0.1
5:3 FTCA	**Average**	**0.4**	**0.3**	**0.3**	**0.3**	**0.3**	**0.3**	**0.3**	**0.3**	**0.4**	**0.5**	**0.4**	**0.5**
	STDEV	0.1	0.0	0.0	0.0	0.0	0.0	0.0	0.0	0.0	0.1	0.1	0.1
4:2FTS	**Average**	**0.1**	**0.1**	**0.1**	**0.1**	**0.1**	**0.1**	**0.1**	**0.2**	**0.1**	**0.1**	**0.1**	**0.1**
	STDEV	0.1	0.1	0.1	0.0	0.1	0.0	0.0	0.0	0.1	0.1	0.1	0.1
6:2FTS	**Average**	**2.3**	**2.0**	**2.6**	**0.3**	**1.1**	**0.5**	**0.5**	**0.5**	**0.3**	**0.5**	**0.4**	**0.4**
	STDEV	0.9	0.5	0.9	0.0	0.2	0.0	0.0	0.0	0.0	0.1	0.0	0.0
8:2FTS	**Average**	**0.2**	**0.2**	**<LOD**	**<LOD**	**<LOD**	**<LOD**	**<LOD**	**<LOD**	**<LOD**	**<LOD**	**<LOD**	**<LOD**

	STDEV	0.0	0.0										
6:2 diPAP	**Average**	**0.1**	**0.2**	**0.1**	**0.2**	**0.2**	**0.4**	**0.3**	**0.4**	**0.6**	**0.5**	**0.4**	**0.5**
	STDEV	0.0	0.1	0.0	0.0	0.0	0.0	0.0	0.1	0.0	0.1	0.1	0.2
PFOSA	**Average**	**0.3**	**0.3**	**0.2**	**0.4**	**0.4**	**0.5**	**0.3**	**0.3**	**0.3**	**0.3**	**0.2**	**0.4**
	STDEV	0.1	0.1	0.0	0.1	0.0	0.0	0.0	0.2	0.1	0.1	0.0	0.2
FBSA	**Average**	**1.1**	**1.0**	**0.8**	**0.7**	**1.0**	**0.6**	**0.9**	**0.5**	**1.1**	**0.6**	**0.3**	**0.2**
	STDEV	0.3	0.0	0.0	0.0	0.2	0.0	0.0	0.0	0.2	0.2	0.1	0.0
FHxSA	**Average**	**0.1**	**0.1**	**0.1**	**0.1**	**0.1**	**0.1**	**0.1**	**<LOD**	**0.1**	**0.1**	**<LOD**	**0.1**
	STDEV	0.1	0.0	0.0	0.0	0.0	0.0	0.0		0.0	0.0		0.0
HFPO-DA	**Average**	**0.1**	**0.1**	**0.2**	**0.1**	**<LOD**	**<LOD**	**0.1**	**<LOD**	**0.1**	**<LOD**	**0.1**	**0.1**
	STDEV	0.1	0.0	0.1	0.0			0.0		0.0		0.0	0.0
PFEESA	**Average**	**0.1**	**0.1**	**0.1**	**0.1**	**<LOD**	**<LOD**	**<LOD**	**<LOD**	**<LOD**	**0.1**	**0.1**	**0.1**
	STDEV	0.0	0.0	0.0	0.0						0.0	0.0	0.0
PF3ONS	**Average**	**<LOD**	**<LOD**	**0.1**	**<LOD**	**<LOD**	**<LOD**	**0.1**	**0.1**	**<LOD**	**<LOD**	**<LOD**	**<LOD**
	STDEV			0.0				0.0	0.0				
**$\sum_{8}$PFCAs**		**21.0**	**21.0**	**19.0**	**19.4**	**19.0**	**18.2**	**21.2**	**22.4**	**19.7**	**18.9**	**18.6**	**20.2**
**$\sum_{7}$PFSAs**		**16.1**	**16.4**	**17.8**	**17.0**	**15.6**	**15.7**	**18.8**	**19.9**	**15.6**	**16.8**	**17.1**	**18.9**
**$\sum_{15}$PFAAs**		**37.0**	**37.4**	**36.8**	**36.4**	**34.6**	**33.8**	**40.0**	**42.3**	**35.2**	**35.6**	**35.7**	**39.0**
**$\sum_{11}$Precursors**		**4.7**	**4.3**	**4.5**	**2.2**	**3.2**	**2.5**	**2.8**	**2.1**	**3.0**	**2.6**	**1.9**	**2.3**

[a] Average value and standard deviation for the duplicate.

[b] Limit of detection based on three times the signal to noise ratio.

Table S6. PFAS concentrations (ng·L^{-1}) in water samples treated by O_3/H_2O_2 AOP at 3.0 mg·L^{-1} O_3 with different H_2O_2 doses.

PFAS	**H_2O_2 dose**	**0 mg·L^{-1}**				**0.05 mg·L^{-1}**				**0.20 mg·L^{-1}**			
	**Time (min)**	**0**	**5**	**10**	**20**	**0**	**5**	**10**	**20**	**0**	**5**	**10**	**20**
PFBA	**Average[a]**	**0.7**	**0.8**	**0.8**	**0.9**	**0.7**	**0.8**	**0.8**	**0.8**	**0.6**	**0.8**	**0.8**	**0.7**
	STDEV[a]	0.0	0.1	0.1	0.0	0.1	0.1	0.1	0.1	0.1	0.1	0.1	0.0
PFPeA	**Average**	**5.2**	**5.0**	**5.2**	**5.3**	**5.2**	**5.1**	**5.2**	**5.2**	**4.8**	**4.9**	**5.0**	**4.9**
	STDEV	0.5	0.1	0.2	0.4	0.4	0.3	0.2	0.6	0.2	0.3	0.2	0.1
PFHxA	**Average**	**7.8**	**7.6**	**7.8**	**7.8**	**7.6**	**7.8**	**7.9**	**7.8**	**7.2**	**7.3**	**7.5**	**7.3**
	STDEV	0.4	2.4	0.4	0.3	0.9	0.1	0.4	0.6	0.6	0.2	1.0	0.5
PFHpA	**Average**	**1.1**	**1.0**	**1.0**	**1.0**	**1.0**	**1.0**	**1.1**	**1.1**	**0.9**	**1.0**	**1.0**	**1.0**
	STDEV	0.7	0.5	0.0	0.1	0.2	0.1	0.1	0.7	0.0	0.1	0.3	0.1
PFOA	**Average**	**4.1**	**3.7**	**3.9**	**4.4**	**4.1**	**3.8**	**3.7**	**4.1**	**3.9**	**3.5**	**3.4**	**3.8**
	STDEV	0.2	0.3	0.6	0.3	0.1	0.2	0.1	0.1	0.3	0.5	0.3	0.2
PFNA	**Average**	**0.8**	**0.6**	**0.7**	**0.9**	**0.7**	**0.6**	**0.6**	**0.8**	**0.7**	**0.6**	**0.6**	**0.7**
	STDEV	0.0	0.0	0.0	0.0	0.0	0.1	0.0	0.2	0.1	0.1	0.1	0.1
PFDA	**Average**	**1.3**	**1.1**	**0.9**	**1.4**	**1.2**	**1.0**	**0.9**	**1.2**	**1.2**	**0.8**	**0.9**	**1.2**
	STDEV	0.2	0.5	0.2	0.0	0.1	0.5	0.0	0.1	0.0	0.1	0.0	0.1
PFUdA	**Average**	**<LOD[b]**	**0.1**	**0.1**	**0.1**	**<LOD**	**0.1**	**<LOD**	**0.1**	**<LOD**	**0.1**	**<LOD**	**<LOD**
	STDEV		0.1	0.0	0.0		0.0		0.0		0.0		
PFPrS	**Average**	**0.1**	**0.2**	**0.2**	**0.2**	**0.1**	**0.2**	**0.1**	**0.1**	**0.2**	**0.1**	**0.1**	**0.1**
	STDEV	0.0	0.0	0.0	0.0	0.0	0.0	0.0	0.1	0.0	0.0	0.0	0.0
PFBS	**Average**	**12.8**	**13.5**	**13.8**	**14.5**	**12.5**	**13.2**	**14.3**	**13.8**	**12.9**	**12.5**	**12.6**	**12.4**
	STDEV	1.3	1.1	1.0	1.3	1.6	1.9	0.9	0.5	0.1	1.0	0.7	0.3
PFPeS	**Average**	**0.1**	**0.1**	**0.1**	**0.1**	**0.1**	**0.1**	**0.1**	**0.1**	**0.1**	**0.1**	**0.1**	**0.1**
	STDEV	0.0	0.0	0.1	0.0	0.0	0.0	0.0	0.0	0.0	0.0	0.0	0.0
PFHxS	**Average**	**0.8**	**0.8**	**0.8**	**0.9**	**0.9**	**0.8**	**0.8**	**0.9**	**0.8**	**0.8**	**0.7**	**0.8**
	STDEV	0.0	0.0	0.1	0.2	0.0	0.0	0.0	0.0	0.0	0.1	0.1	0.0
PFHpS	**Average**	**0.1**	**0.3**	**0.3**	**0.4**	**0.1**	**0.2**	**0.2**	**0.3**	**0.1**	**0.2**	**0.2**	**0.2**
	STDEV	0.0	0.0	0.1	0.1	0.0	0.0	0.0	0.0	0.0	0.0	0.0	0.1
PFOS	**Average**	**2.2**	**2.1**	**2.0**	**4.4**	**2.3**	**1.8**	**1.8**	**4.4**	**2.0**	**4.0**	**1.5**	**2.1**
	STDEV	0.6	0.9	0.1	0.1	0.3	0.1	0.0	0.0	0.1	0.2	0.3	0.8
PFNS	**Average**	**<LOD**	**1.1**	**1.1**	**1.0**	**<LOD**	**0.6**	**0.6**	**0.5**	**<LOD**	**0.3**	**0.3**	**0.4**
	STDEV		0.2	0.4	0.2		0.0	0.0	0.0		0.0	0.0	0.0
5:3 FTCA	**Average**	**0.8**	**0.4**	**0.6**	**0.8**	**0.6**	**2.7**	**2.1**	**1.0**	**0.6**	**0.5**	**0.7**	**0.2**
	STDEV	0.1	0.1	0.1	0.0	0.2	1.0	0.6	0.2	0.1	0.1	0.1	0.0
4:2FTS	**Average**	**0.2**	**0.1**	**0.1**	**0.1**	**0.2**	**0.1**	**0.1**	**0.1**	**0.2**	**0.1**	**0.1**	**0.1**
	STDEV	0.0	0.0	0.0	0.0	0.0	0.1	0.1	0.0	0.1	0.0	0.1	0.1
6:2FTS	**Average**	**0.2**	**0.9**	**0.5**	**0.4**	**0.2**	**0.3**	**0.5**	**0.4**	**0.2**	**0.3**	**0.3**	**0.3**
	STDEV	0.0	0.1	0.0	0.0	0.0	0.1	0.1	0.1	0.0	0.1	0.1	0.0
8:2FTS	**Average**	**<LOD**	**<LOD**	**<LOD**	**<LOD**	**<LOD**	**<LOD**	**<LOD**	**<LOD**	**<LOD**	**<LOD**	**<LOD**	**<LOD**

	STDEV												
6:2 diPAP	**Average**	**0.2**	**0.4**	**0.1**	**0.5**	**0.1**	**0.4**	**0.4**	**0.2**	**0.2**	**0.2**	**0.2**	**0.1**
	STDEV	0.0	0.0	0.0	0.0	0.0	0.1	0.1	0.0	0.0	0.0	0.1	0.0
PFOSA	**Average**	**0.3**	**0.3**	**0.1**	**0.3**	**0.3**	**0.1**	**0.3**	**0.2**	**0.2**	**0.3**	**0.2**	**0.3**
	STDEV	0.0	0.0	0.1	0.0	0.0	0.0	0.1	0.1	0.1	0.0	0.1	0.0
FBSA	**Average**	**0.6**	**<LOD**	**0.1**	**<LOD**	**0.2**	**<LOD**	**<LOD**	**<LOD**	**0.6**	**0.1**	**0.1**	**0.7**
	STDEV	0.0		0.0		0.1				0.1	0.1	0.0	0.1
FHxSA	**Average**	**<LOD**	**<LOD**	**<LOD**	**<LOD**	**<LOD**	**<LOD**	**<LOD**	**<LOD**	**<LOD**	**0.1**	**0.1**	**0.1**
	STDEV										0.0	0.1	0.0
HFPO-DA	**Average**	**<LOD**	**<LOD**	**<LOD**	**<LOD**	**<LOD**	**<LOD**	**<LOD**	**<LOD**	**<LOD**	**<LOD**	**<LOD**	**<LOD**
	STDEV												
PFEESA	**Average**	**0.1**	**0.1**	**0.1**	**0.1**	**0.1**	**0.1**	**0.1**	**0.1**	**0.1**	**0.1**	**0.1**	**0.1**
	STDEV	0.0	0.0	0.0	0.0	0.0	0.0	0.0	0.0	0.0	0.0	0.0	0.0
PF3ONS	**Average**	**<LOD**	**<LOD**	**<LOD**	**<LOD**	**<LOD**	**<LOD**	**<LOD**	**<LOD**	**<LOD**	**<LOD**	**<LOD**	**0.1**
	STDEV												0.0
**$\sum_8$PFCAs**		**20.9**	**19.9**	**20.3**	**21.7**	**20.5**	**20.2**	**20.1**	**21.2**	**19.4**	**19.0**	**19.0**	**19.7**
**$\sum_7$PFSAs**		**16.1**	**18.0**	**18.3**	**21.3**	**16.0**	**16.8**	**17.9**	**20.1**	**16.0**	**17.9**	**15.5**	**16.1**
**$\sum_{15}$PFAAs**		**37.0**	**37.9**	**38.6**	**43.1**	**36.5**	**37.1**	**38.0**	**41.3**	**35.3**	**36.9**	**34.5**	**35.8**
**$\sum_9$Precursors**		**2.3**	**2.2**	**1.7**	**2.2**	**1.6**	**3.7**	**3.4**	**2.1**	**2.1**	**1.7**	**1.8**	**2.0**

[a] Average value and standard deviation for the duplicate.

[b] Limit of detection based on three times the signal to noise ratio.

Table S7. PFAS concentrations (ng·L^{-1}) after TOP oxidation in water samples treated by various ozone/H_2O_2 doses.

PFAS	H_2O_2 dose	0 mg·L^{-1}				0.05 mg·L^{-1}				0.20 mg·L^{-1}			
	Time (min)	0	5	10	20	0	5	10	20	0	5	10	20
PFBA	Average[a]	1.0	1.0	1.5	1.2	1.6	1.2	1.1	0.8	1.4	1.0	1.0	0.9
	STDEV[a]	0.1	0.0	0.5	0.6	0.2	0.0	0.0	0.2	0.8	0.6	0.0	0.2
PFPeA	Average	4.4	4.2	4.0	4.3	3.4	3.3	3.5	3.6	4.0	4.2	3.8	3.6
	STDEV	0.4	1.0	0.8	1.2	0.8	0.8	0.1	0.0	0.1	0.2	1.0	0.2
PFHxA	Average	7.8	8.4	8.7	8.1	6.8	7.6	6.5	6.5	7.8	7.7	7.5	7.3
	STDEV	0.2	1.0	1.9	0.3	0.1	0.9	0.9	0.5	0.6	0.4	0.3	0.2
PFHpA	Average	1.3	1.0	1.4	0.8	1.1	1.0	0.9	0.7	1.5	1.1	1.1	1.3
	STDEV	0.7	0.1	0.0	0.4	0.3	0.0	0.0	0.0	0.3	0.3	0.0	0.4
PFOA	Average	16.9	23.6	20.6	16.7	15.4	14.4	10.6	12.6	10.3	7.0	8.3	11.5
	STDEV	1.2	1.2	5.7	0.8	5.3	1.6	1.1	2.2	1.0	0.1	0.8	0.8
PFNA	Average	0.9	0.9	0.7	1.1	0.6	0.5	0.7	0.9	0.6	0.7	0.7	0.7
	STDEV	0.4	0.0	0.1	0.8	0.0	0.1	0.1	0.2	0.1	0.0	0.0	0.1
PFDA	Average	3.7	4.8	3.9	2.6	3.2	3.3	3.6	3.4	5.1	3.4	3.6	3.4
	STDEV	0.1	1.0	0.5	0.4	0.2	0.7	0.2	0.2	0.1	0.6	0.1	0.2
PFUdA	Average	0.4	0.7	0.8	0.8	0.4	0.4	0.4	0.5	0.7	0.6	0.9	0.4
	STDEV	0.1	0.0	1.1	0.6	0.1	0.0	0.0	0.2	0.8	0.2	0.8	0.0
PFPrS	Average	0.2	0.2	0.2	0.1	0.2	0.3	0.2	0.2	0.2	0.1	0.2	0.2
	STDEV	0.1	0.0	0.1	0.0	0.0	0.0	0.0	0.0	0.1	0.0	0.0	0.0
PFBS	Average	17.8	16.8	21.1	18.7	13.4	16.5	18.3	17.6	16.6	17.4	16.8	16.8
	STDEV	1.0	2.1	3.7	0.1	2.5	2.1	1.1	1.4	2.3	2.6	1.7	1.6
PFPeS	Average	0.1	0.1	0.1	0.1	0.1	0.1	0.1	0.1	0.1	0.0	0.1	0.1
	STDEV	0.0	0.0	0.0	0.0	0.0	0.0	0.0	0.0	0.0	0.0	0.0	0.0
PFHxS	Average	0.8	0.6	0.9	0.7	0.7	0.7	0.6	0.7	0.7	0.6	0.6	0.7
	STDEV	0.2	0.1	0.1	0.1	0.2	0.0	0.0	0.0	0.0	0.0	0.0	0.1
PFHpS	Average	0.1	0.1	0.1	0.1	0.1	0.1	0.1	0.1	0.1	0.1	0.1	0.1
	STDEV	0.0	0.0	0.0	0.0	0.0	0.0	0.0	0.0	0.0	0.0	0.0	0.0
PFOS	Average	1.2	1.5	1.6	2.1	1.6	1.8	2.9	2.4	3.4	1.9	2.5	2.2
	STDEV	0.1	0.1	0.3	0.2	0.4	0.0	0.9	0.2	0.1	0.6	0.5	0.6
PFNS	Average	<LOD[b]	<LOD	<LOD	0.2	<LOD	<LOD	<LOD	0.1	<LOD	<LOD	<LOD	<LOD
	STDEV				0.0				0.1				
5:3 FTCA	Average	<LOD	<LOD	<LOD	<LOD	<LOD	<LOD	<LOD	<LOD	<LOD	<LOD	<LOD	<LOD
	STDEV												
4:2FTS	Average	0.3	0.2	0.4	0.4	0.1	0.1	0.1	0.1	0.2	0.3	0.2	0.1
	STDEV	0.1	0.0	0.1	0.1	0.1	0.0	0.0	0.0	0.1	0.1	0.0	0.1
6:2FTS	Average	0.3	0.0	0.3	0.2	0.4	0.5	0.4	0.2	0.2	0.3	0.2	0.2
	STDEV	0.0	0.0	0.0	0.0	0.1	0.0	0.0	0.0	0.0	0.0	0.0	0.0
8:2FTS	Average	<LOD	<LOD	<LOD	<LOD	<LOD	<LOD	<LOD	<LOD	<LOD	<LOD	<LOD	<LOD

	STDEV												
6:2 diPAP	**Average**	**0.2**	**1.2**	**0.6**	**0.7**	**0.2**	**1.0**	**0.9**	**1.4**	**1.5**	**0.8**	**1.7**	**0.8**
	STDEV												
PFOSA	**Average**	**0.3**	**0.2**	**0.2**	**0.3**	**0.2**	**0.2**	**0.1**	**0.2**	**0.3**	**0.2**	**0.2**	**0.2**
	STDEV	0.0	0.0	0.0	0.0	0.1	0.0	0.0	0.0	0.0	0.1	0.1	0.0
FBSA	**Average**	**<LOD**	**<LOD**	**<LOD**	**0.1**	**0.3**	**<LOD**	**<LOD**	**<LOD**	**<LOD**	**<LOD**	**<LOD**	**<LOD**
	STDEV				0.0	0.1							
FHxSA	**Average**	**<LOD**	**<LOD**	**<LOD**	**<LOD**	**<LOD**	**<LOD**	**<LOD**	**<LOD**	**1.6**	**<LOD**	**0.1**	**0.1**
	STDEV									0.3		0.0	0.0
HFPO-DA	**Average**	**<LOD**	**0.1**	**<LOD**	**<LOD**	**<LOD**	**<LOD**	**<LOD**	**<LOD**	**<LOD**	**<LOD**	**<LOD**	**<LOD**
	STDEV		0.0										
PFEESA	**Average**	**0.1**	**0.7**	**<LOD**	**<LOD**	**<LOD**	**<LOD**	**<LOD**	**<LOD**	**<LOD**	**<LOD**	**<LOD**	**<LOD**
	STDEV	0.0	0.1										
PF3ONS	**Average**	**0.3**	**0.7**	**0.5**	**0.3**	**0.1**	**0.2**	**0.1**	**0.1**	**0.1**	**0.1**	**0.1**	**0.1**
	STDEV	0.0	0.0	0.1	0.0	0.0	0.0	0.0	0.0	0.0	0.1	0.0	0.0
**$\sum_8$PFCAs**		**36.5**	**44.5**	**41.5**	**35.7**	**32.4**	**31.7**	**27.4**	**29.1**	**31.6**	**25.6**	**27.0**	**29.3**
**$\sum_7$PFSAs**		**20.1**	**19.4**	**24.0**	**22.1**	**16.1**	**19.3**	**22.1**	**21.2**	**21.0**	**20.1**	**20.3**	**20.1**
**$\sum_{15}$PFAAs**		**56.5**	**63.9**	**65.5**	**57.8**	**48.5**	**51.0**	**49.5**	**50.3**	**52.6**	**45.7**	**47.4**	**49.3**
**$\sum_9$Precursors**		**1.5**	**3.1**	**1.9**	**1.9**	**1.3**	**1.9**	**1.6**	**2.1**	**3.9**	**1.7**	**2.6**	**1.6**

[a] Average value and standard deviation for the duplicate.

[b] Limit of detection based on three times the signal to noise ratio.

Table S8. The increase of PFAA concentrations (ΔPFAA, in ng·L^{-1}) after TOP oxidation in water samples treated by various ozone/H_2O_2 doses.

**ΔPFAA[a]**	**H_2O_2 dose**	**0 mg·L^{-1}**				**0.05 mg·L^{-1}**				**0.20 mg·L^{-1}**			
	**Time (min)**	**0**	**5**	**10**	**20**	**0**	**5**	**10**	**20**	**0**	**5**	**10**	**20**
PFBA		0.3	0.2	0.7	0.3	0.9	0.4	0.3	0.0	0.7	0.3	0.2	0.2
PFHxA		0.1	0.9	0.9	0.2	0.0	0.0	0.0	0.0	0.7	0.3	0.1	0.0
PFHpA		0.2	0.0	0.4	0.0	0.0	0.0	0.0	0.0	0.6	0.1	0.1	0.3
PFOA		12.8	19.9	16.7	12.4	11.4	10.7	6.9	8.5	6.4	3.6	4.9	7.7
PFNA		0.1	0.3	0.0	0.3	0.0	0.0	0.1	0.0	0.0	0.0	0.2	0.0
PFDA		2.4	3.7	3.0	1.3	2.0	2.2	2.8	2.2	3.9	2.5	2.8	2.3
PFUdA		0.4	0.6	0.7	0.7	0.4	0.3	0.4	0.5	0.8	0.5	0.9	0.4
PFPrS		0.1	0.0	0.0	0.0	0.1	0.1	0.1	0.1	0.1	0.0	0.1	0.1
PFBS		5.0	3.3	7.3	4.3	0.9	3.3	3.9	3.8	3.7	4.9	4.2	4.4
PFPeS		0.0	0.1	0.0	0.0	0.0	0.0	0.0	0.0	0.0	0.0	0.0	0.0
PFHxS		0.0	0.0	0.1	0.0	0.0	0.0	0.0	0.0	0.0	0.0	0.0	0.0
PFHpS		0.0	0.0	0.0	0.0	0.0	0.0	0.0	0.0	0.0	0.0	0.0	0.0
PFOS		0.0	0.0	0.0	0.0	0.0	0.0	1.1	0.0	1.4	0.0	1.0	0.1
**∑PFCAs**		**16.3**	**25.5**	**22.4**	**15.2**	**14.7**	**13.6**	**10.6**	**11.2**	**13.1**	**7.3**	**9.2**	**10.9**
**∑PFSAs**		**5.1**	**3.4**	**7.4**	**4.3**	**1.0**	**3.4**	**5.1**	**3.9**	**5.1**	**4.9**	**5.4**	**4.6**

[a] Average value of the difference of PFAA concentrations before/after TOP oxidation.

Table S9. Statistical analysis between contact time (t in min) and classified/individual PFAS concentrations (c_{PFAS} in ng·L^{-1}) in water samples treated by ozonation and ozone/H_2O_2 AOP processes, showing correlation analysis and liner regressions results.

Treatment	Variable	Correlation coefficients	t-stat	p-value
O_3 = 3.0 mg·L^{-1}	c_{PFSAs}	0.9431	4.01	0.057
	c_{PFSAs} = 0.223 t + 16.79 (adjusted R^2 = 0.8341)			
O_3 = 3.0 mg·L^{-1}	c_{PFBS}	0.9697	5.62	0.030
	c_{PFBS} = 0.080 t + 12.97 (adjusted R^2 = 0.9105)			
O_3 = 4.0 mg·L^{-1}	c_{PFSAs}	0.9986	28.30	1.25×10^{-3}
	c_{PFSAs} = 0.154 t + 15.55 (adjusted R^2 = 0.9963)			
O_3 = 4.0 mg·L^{-1}	c_{PFBS}	0.9999	134.84	5.50×10^{-5}
	c_{PFBS} = 0.112 t + 12.39 (adjusted R^2 = 0.9998)			
H_2O_2 = 0.05 mg·L^{-1}	c_{PFSAs}	0.9984	25.08	1.59×10^{-3}
	c_{PFSAs} = 0.205 t + 15.91 (adjusted R^2 = 0.9953)			

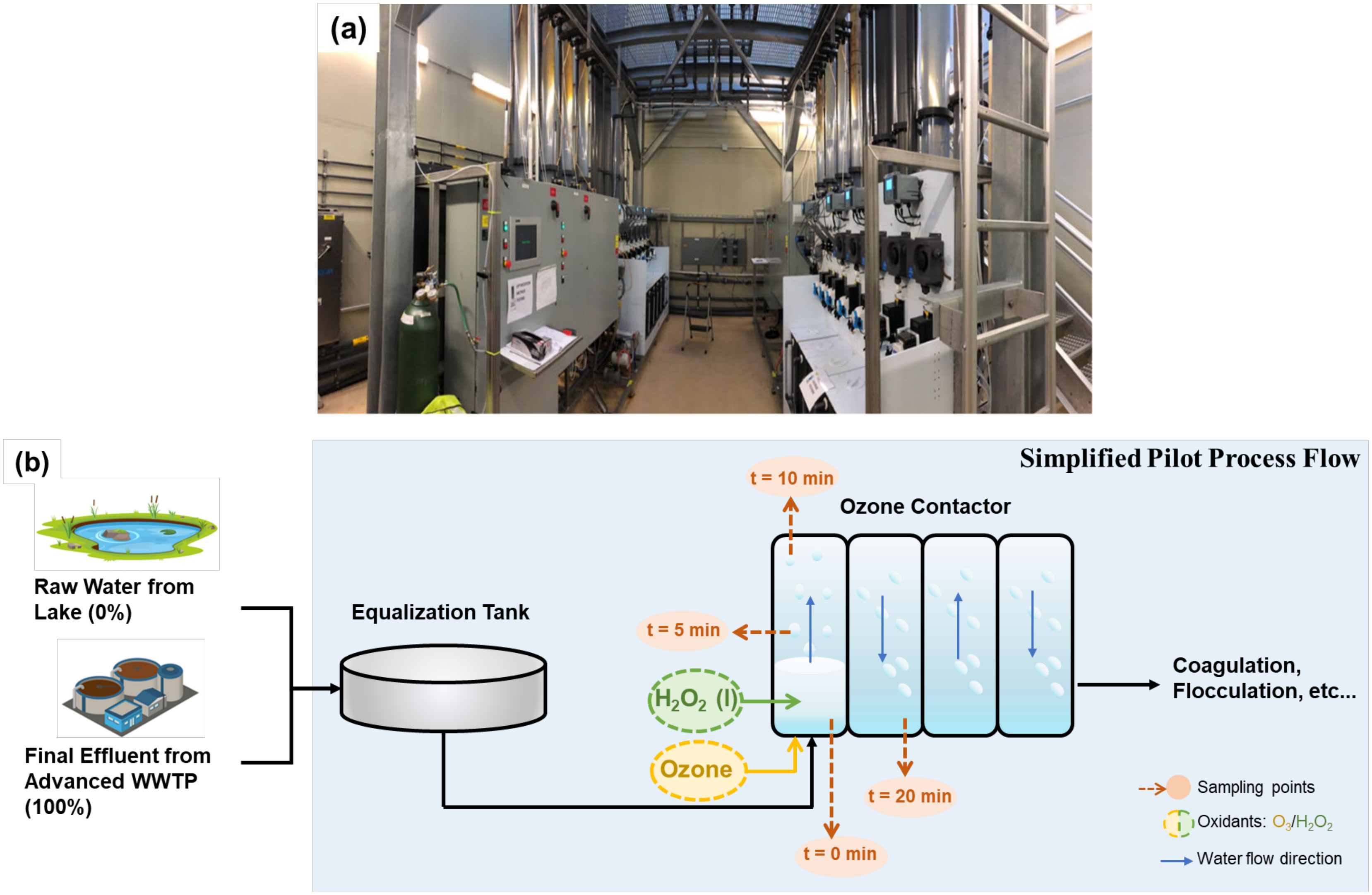

Figure S1. (a) Photo of pilot plant for testing. (b) Schematic diagrams of the treatment processes at pilot plant.

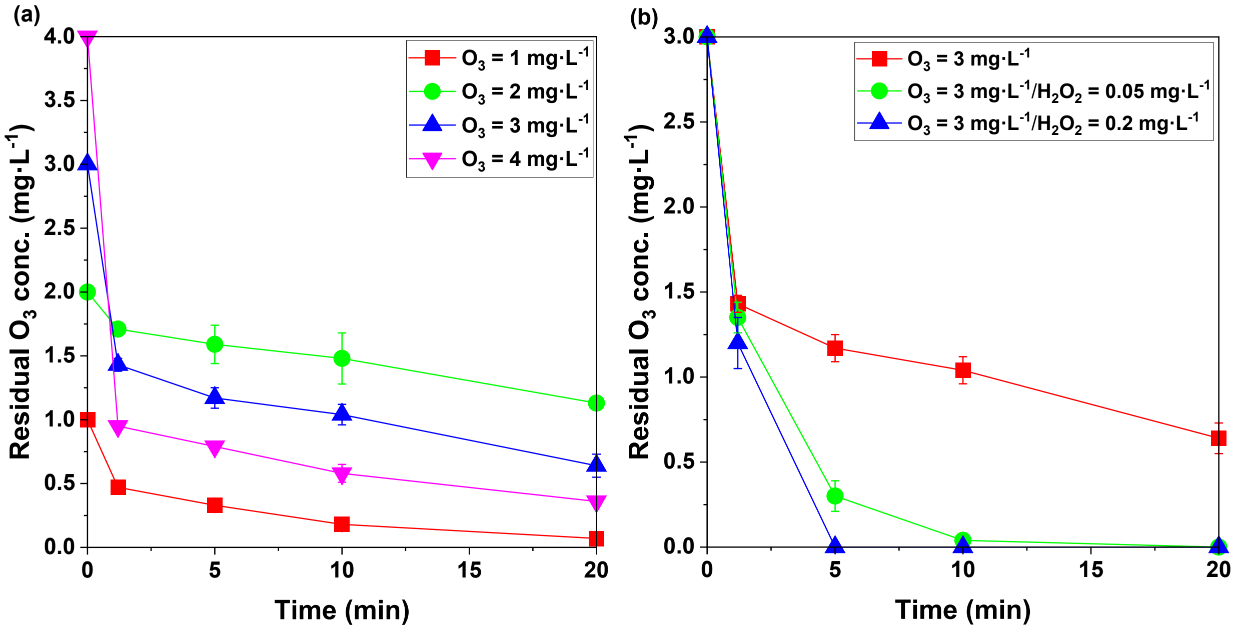

Figure S2. Residual ozone concentrations (mg·L^{-1}) in (a) water samples treated by various ozone doses; (b) water samples treated by various H_2O_2 doses with an initial O_3 dose = 3.0 mg·L^{-1}.

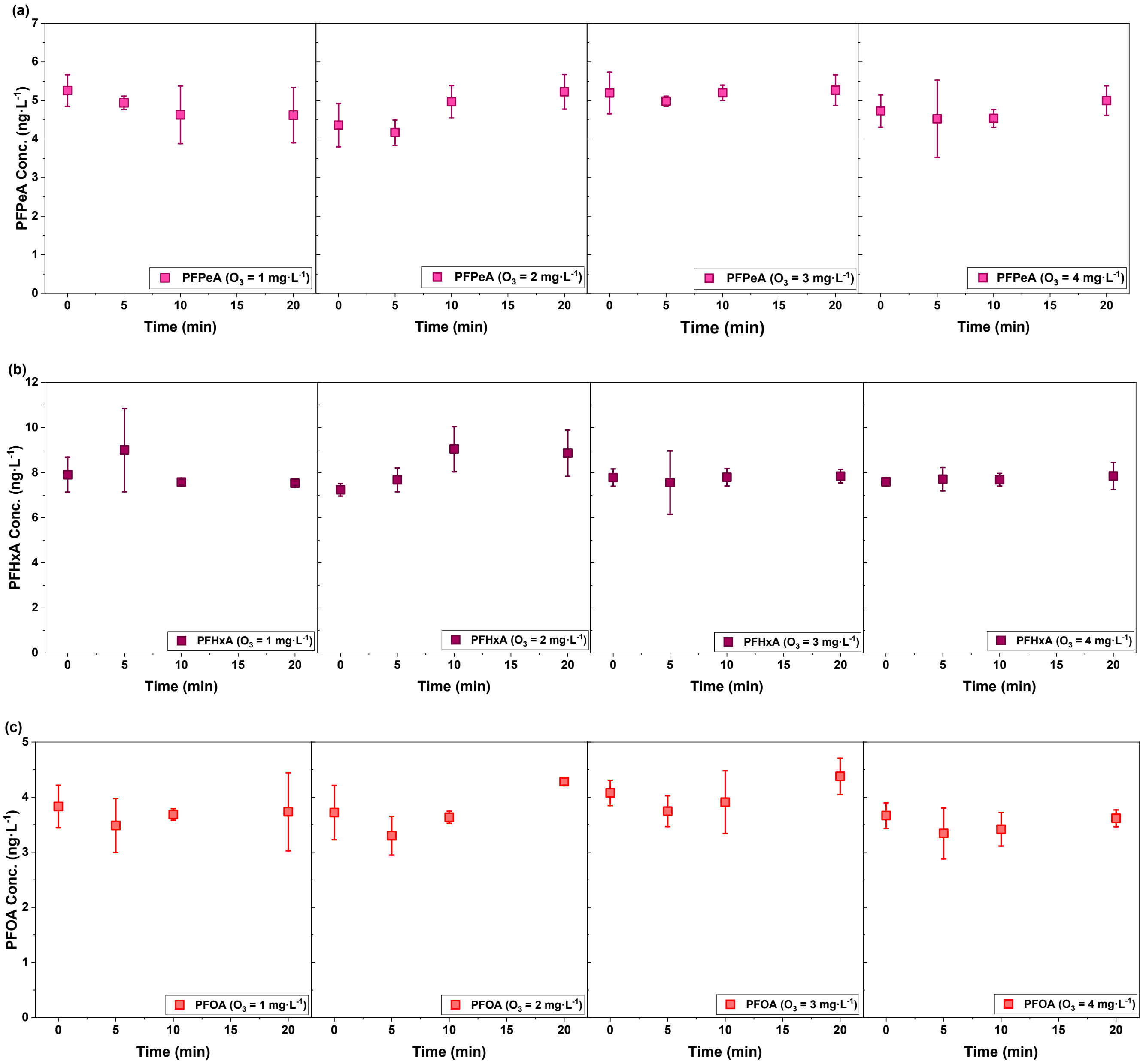

(a)
PFPeA Conc. (ng·L-1)
PFPeA (O3 = 1 mg·L-1)
PFPeA (O3 = 2 mg·L-1)
PFPeA (O3 = 3 mg·L-1)
PFPeA (O3 = 4 mg·L-1)
Time (min)
(b)
PFHxA Conc. (ng·L-1)
PFHxA (O3 = 1 mg·L-1)
PFHxA (O3 = 2 mg·L-1)
PFHxA (O3 = 3 mg·L-1)
PFHxA (O3 = 4 mg·L-1)
Time (min)
(c)
PFOA Conc. (ng·L-1)
PFOA (O3 = 1 mg·L-1)
PFOA (O3 = 2 mg·L-1)
PFOA (O3 = 3 mg·L-1)
PFOA (O3 = 4 mg·L-1)
Time (min)

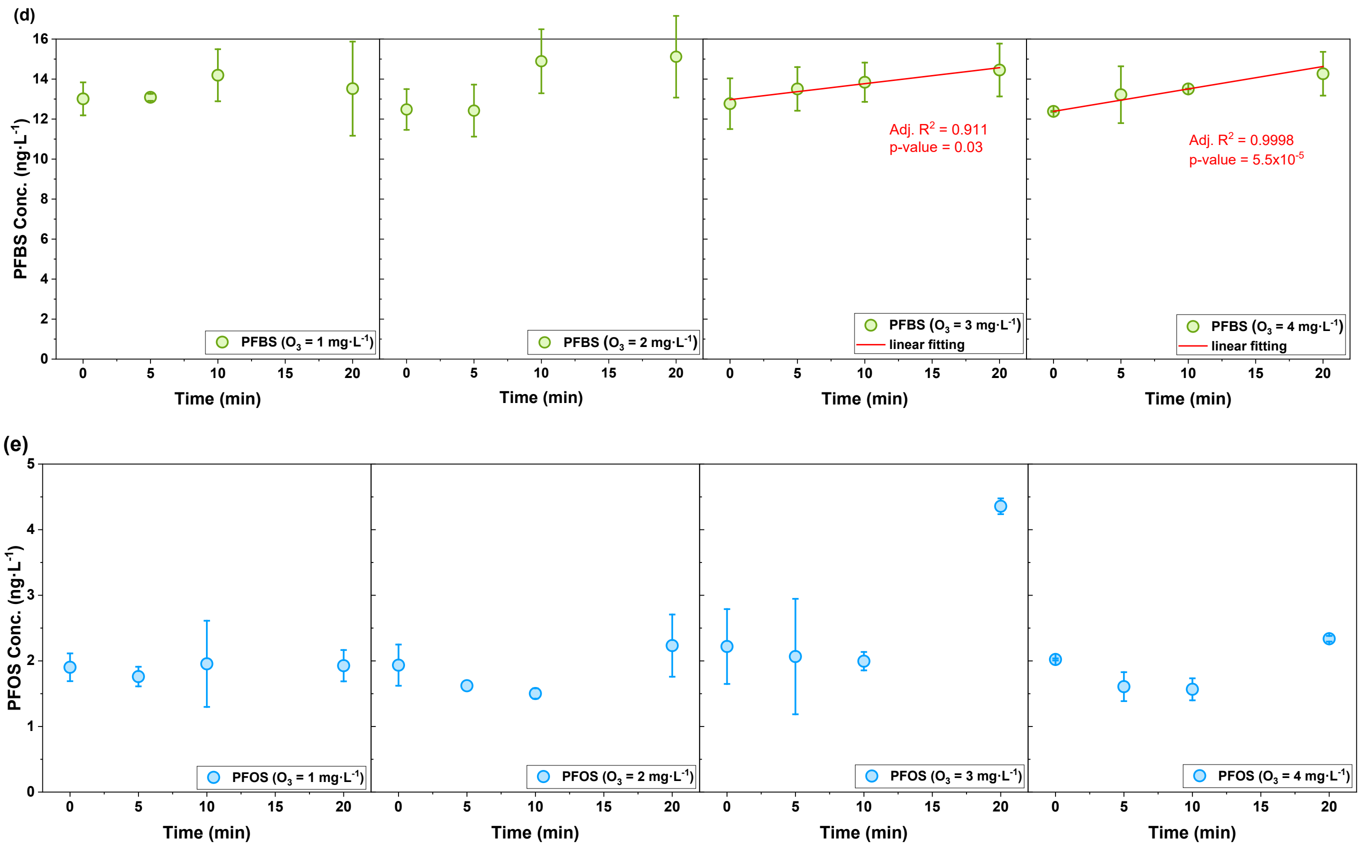

Figure S3. Averaged concentrations of representative individual PFAA (ng·L^{-1}) in ozone-treated water samples: (a) PFPeA; (b) PFHxA; (c) PFOA; (d) PFBS; (e) PFOS.

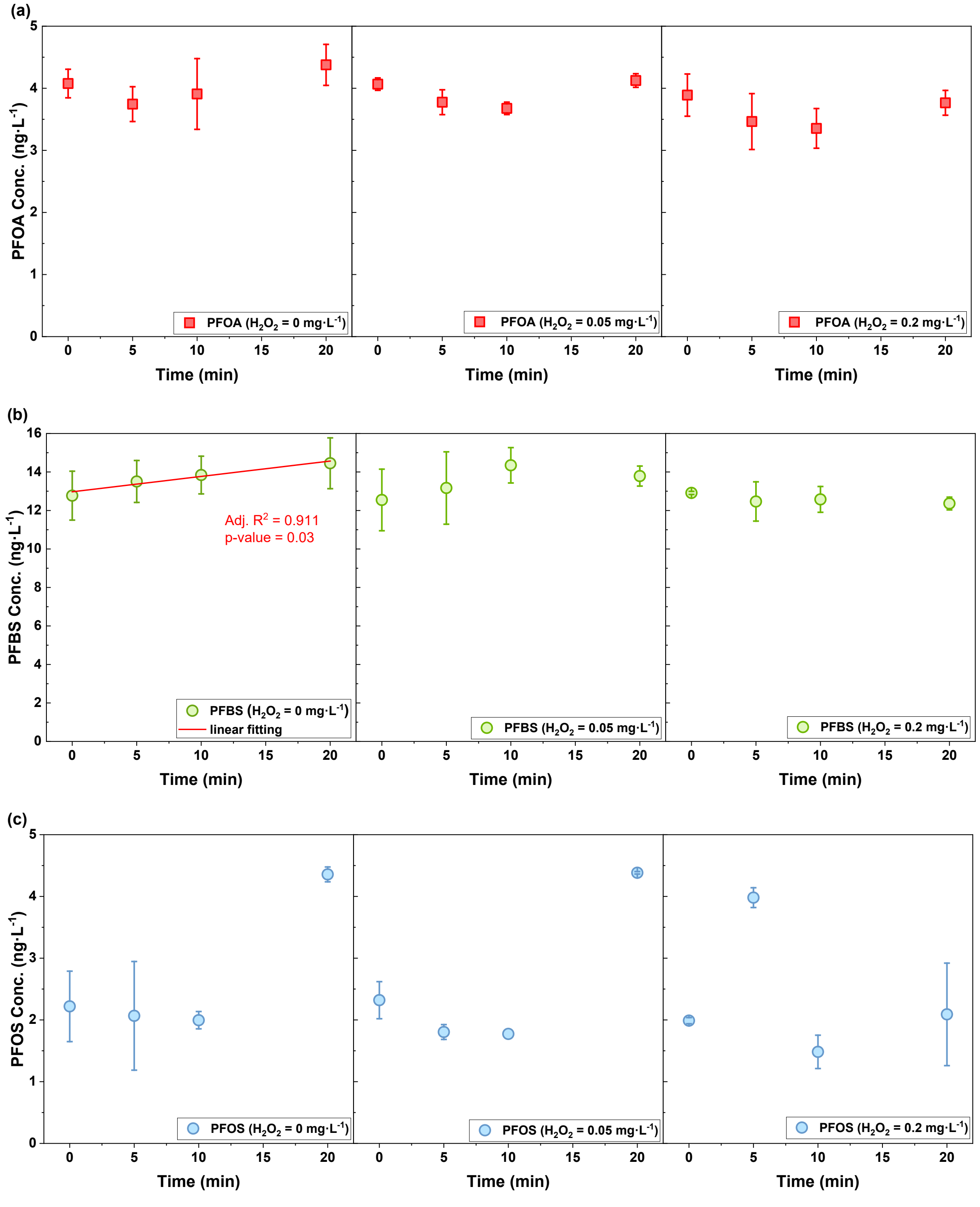

Figure S4. Averaged concentrations of representative individual PFAA ($ng\cdot L^{-1}$) in ozone/H_2O_2 AOP-treated water samples: (a) PFOA; (b) PFBS; (c) PFOS.

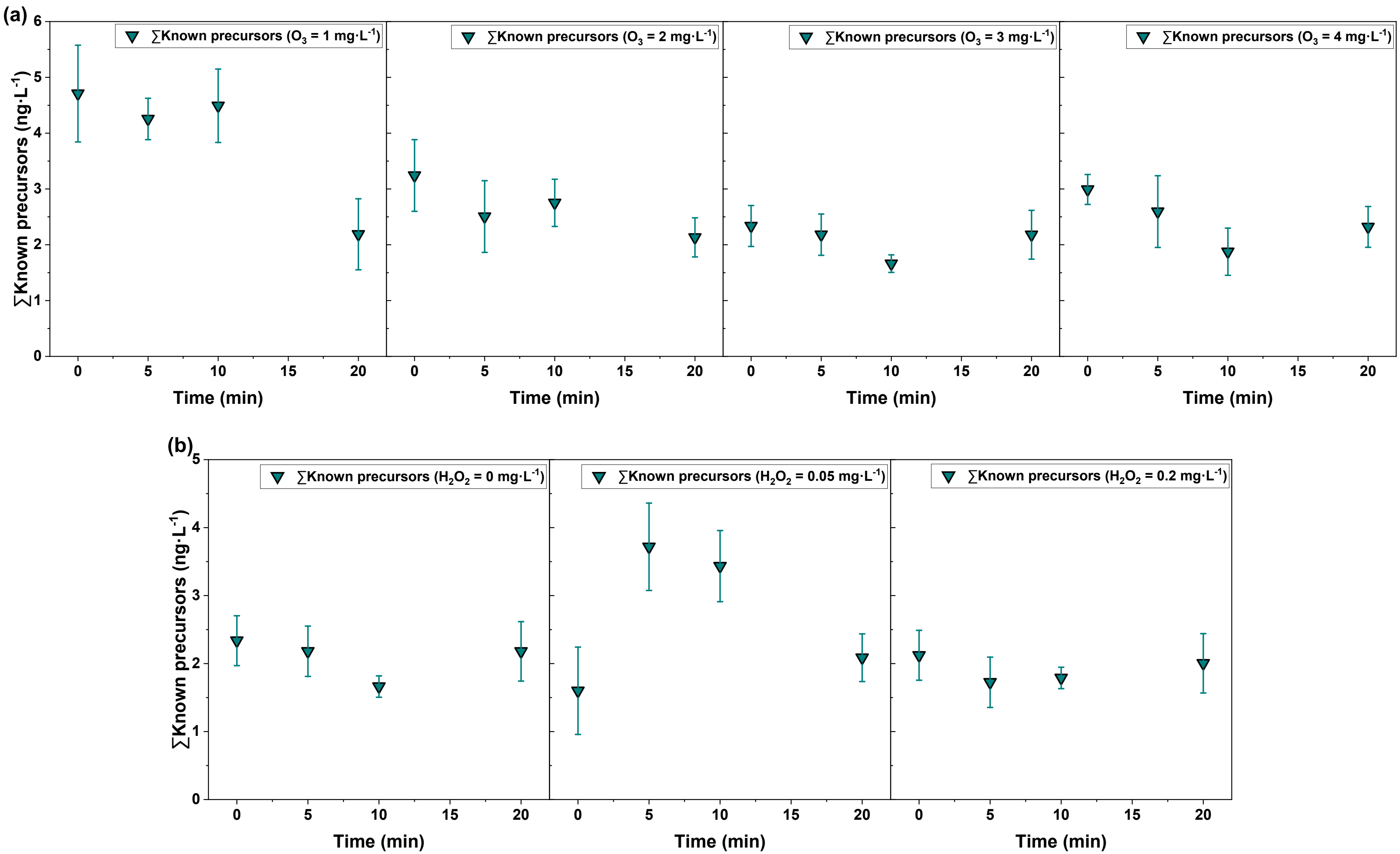

Figure S5. Averaged concentrations of known PFAS precursors ($ng \cdot L^{-1}$) in ozone-treated and ozone/H_2O_2 AOP-treated water samples.
